# An *in silico* cortico-basal ganglia-thalamic testbed for comparing visual and deep brain stimulation in Parkinson’s disease

**DOI:** 10.3389/fncom.2026.1950516

**Published:** 2026-10-02

**Authors:** Chun-Hui Huang, Wen-Shuo Chen, Jin-Rong Lai, Chia-Hung Cho, Yun-Ta Sung, Chih-Tsung Chang, Chii-Wann Lin

**Affiliations:** 1 Department of Biomedical Engineering, National Taiwan University, Taipei, Taiwan; 2 Graduate Institute of Biomedical Electronics and Bioinformatics, National Taiwan University, Taipei, Taiwan; 3 Department of Electrical Engineering, National Taiwan Ocean University, Keelung, Taiwan; 4 Institute of Biomedical Engineering and Nanomedicine, National Health Research Institutes, Zhunan, Taiwan

**Keywords:** computational testbed, cortico-basal ganglia-thalamic loop, deep brain stimulation, Hodgkin-Huxley model, neuromodulation, Parkinson’s disease, periodic visual input

## Abstract

**Introduction:**

Deep brain stimulation (DBS) is an established intervention for Parkinson’s disease (PD), whereas the network-level effects of prescribed periodic visual input implemented with 10 Hz base pulses remain uncertain.

**Methods:**

We developed a conductance-based cortico-basal ganglia-thalamic (CBT) model comprising 11 Hodgkin-Huxley neuronal populations and a prescribed periodic visual-input unit (RET) to compare 130 Hz subthalamic nucleus (STN) DBS with the archived periodic visual-input condition on the same calibrated PD network. Primary conditions used *N* = 10 independent runs each. Additional analyses included an unpaired 0–5 mA/cm^2^ DBS-amplitude series, a matched-seed internal-globus-pallidus-to-thalamocortical-relay (GPi→TC) sensitivity analysis at 0.8, 1.0, and 1.2 × nominal coupling (*N* = 10 per scale), and a matched-seed three-parameter ±20% local sensitivity analysis (*N* = 5 per parameter-condition cell).

**Results:**

DBS reduced the model-internal STN synaptic-current beta measure from 0.0184 ± 0.0060 to 0.0018 ± 0.0004 a.u. (Holm-corrected *p* = 1.1 × 10^–5^), whereas periodic visual input increased thalamocortical relay (TC) and reticular thalamic (RE) firing and enhanced cortical pyramidal (PY) gamma (30–80 Hz) and narrowband 38–42 Hz power. Cortical PY firing was 4.86 ± 0.84 Hz in PD, 5.28 ± 0.87 Hz with DBS (*p* = 0.285 versus PD), and 10.33 ± 0.85 Hz with periodic visual input (Holm-corrected *p* = 1.18 × 10^–10^ versus PD). The ampltude series showed a non-monotonic STN response at 1 mA/cm^2^; from 2 to 5 mA/cm^2^, mean STN beta values occupied a narrow range of 0.00186–0.00212 a.u. In the GPi→TC analysis, TC spontaneous firing remained 0 Hz in 30/30 runs, and all 750,000 saved voltage samples were finite and subthreshold (–72.06 to –54.74 mV; no 0-mV crossings). Across the three-parameter analysis, DBS raw STN-beta suppression remained 88.9%–95.8%, and periodic visual input increased TC firing 3.70–4.29-fold relative to nominal PD.

**Discussion:**

These findings show persistence across the tested dose, GPi→TC, and three-parameter local domains but do not establish evoked relay fidelity, global parameter robustness, biological mechanisms, or clinical efficacy.

## 1 Introduction

Parkinson’s disease (PD) is a progressive neurodegenerative disorder in which loss of nigrostriatal dopamine reorganizes direct- and indirect-pathway activity and promotes abnormal synchronization across the cortico-basal ganglia-thalamic (CBT) network (McGregor and Nelson, 2019; Zhai et al., 2019). Excessive beta-band activity (13–30 Hz) is associated with rigidity and bradykinesia and provides a useful, although not exclusive, circuit-level marker of the parkinsonian state (Little and Brown, 2012; Mallet et al., 2019).

Levodopa remains the principal pharmacological treatment, but long-term use can be complicated by motor fluctuations and dyskinesia (Jankovic and Aguilar, 2008). For advanced PD, high-frequency deep brain stimulation (DBS) of the subthalamic nucleus (STN) is an established surgical option that changes basal-ganglia output and suppresses pathological beta activity (Herrington et al., 2016). Rhythmic sensory stimulation has also been investigated as a non-invasive way to influence distributed neural activity; 40 Hz visual stimulation can entrain gamma-band responses and recruit higher-order regions in experimental settings (Adaikkan et al., 2019); feasibility of 40 Hz trans-eyelid stimulation with steady-state visual-evoked-potential recording has also been reported in healthy adults (Huang et al., 2026). These 40-Hz studies provide broader context for temporally patterned sensory stimulation but do not specify the frequency implemented in the present model.

Computational descriptions of PD span complementary levels. Conductance-based and spiking models resolve population and synaptic dynamics. In contrast, cortex-basal ganglia neural-mass models use Hopf-bifurcation analysis to identify how coupling and heterogeneous or distributed delays generate beta oscillations (Wang et al., 2022, 2023a). Models incorporating direct basal-ganglia feedback to cortex further show that feedback strength and delay can shift oscillatory regimes (Wang et al., 2023b). These approaches motivate explicit sensitivity and scope statements when interpreting any single calibrated network.

Here we integrate cortex, thalamus, striatum, and basal ganglia into a reconfigurable Hodgkin-Huxley CBT testbed and compare 130 Hz STN-DBS with a prescribed periodic visual input implemented with 10 Hz base pulses on the same PD network. We evaluate subcortical spectral measures together with thalamic and cortical firing and spectral outputs. The visual pathway is deliberately represented by a prescribed periodic visual-input unit (RET) rather than an explicit lateral geniculate nucleus (LGN), and the head-to-head intervention comparison is restricted to the awake PD state. The aim is therefore to generate quantitatively testable model hypotheses, not to establish a clinical treatment principle. The archived executable implements the visual condition as a 10 Hz base-pulse train; the internal identifier PD_visual40 is retained only for file compatibility, and 38–42 Hz is analyzed as an output spectral band rather than asserted as the input frequency. Accordingly, the present simulations should not be interpreted as a computational implementation of 40-Hz sensory entrainment.

## 2 Materials and methods

### 2.1 Integrative CBT network modeling

The model couples microscopic ion-channel dynamics with circuit topology to provide a common computational substrate for testing state and stimulation perturbations.

#### 2.1.1 Hodgkin-Huxley neuron formalism

Following the Hodgkin-Huxley formalism (Hodgkin and Huxley, 1952), the CBT model comprises 11 neuronal populations spanning cortex, thalamus, striatum, and basal ganglia: cortical pyramidal neurons (PY), low-threshold-spiking interneurons (LTS), cortical fast-spiking interneurons (cFS), thalamocortical relay cells (TC), reticular thalamic neurons (RE), striatal fast-spiking interneurons (sFS), direct-pathway medium spiny neurons (dMSN), indirect-pathway medium spiny neurons (iMSN), external and internal globus pallidus neurons (GPe and GPi), and subthalamic nucleus neurons. Following Ching et al. (2010), the cortical module contains two structurally identical areas, each with 40 PY, 10 LTS, and 10 cFS neurons; both receive the prescribed RET drive and project symmetrically to the striatal populations. Thus, the model contains 80 PY, 20 LTS, and 20 cFS neurons in total, and each thalamic or basal-ganglia population contains 10 neurons. RET is a prescribed periodic visual-input unit that delivers fixed 10 Hz base-pulse trains to PY and TC. RET has no Hodgkin-Huxley membrane dynamics and is treated as an input unit rather than as a twelfth neuronal population. The membrane potential V(t) of each modeled neuron follows the Kirchhoff current-balance relationship given in Equation 1 (full formulations in Supplementary Table 1; network topology in Figure 1):

**FIGURE 1:**
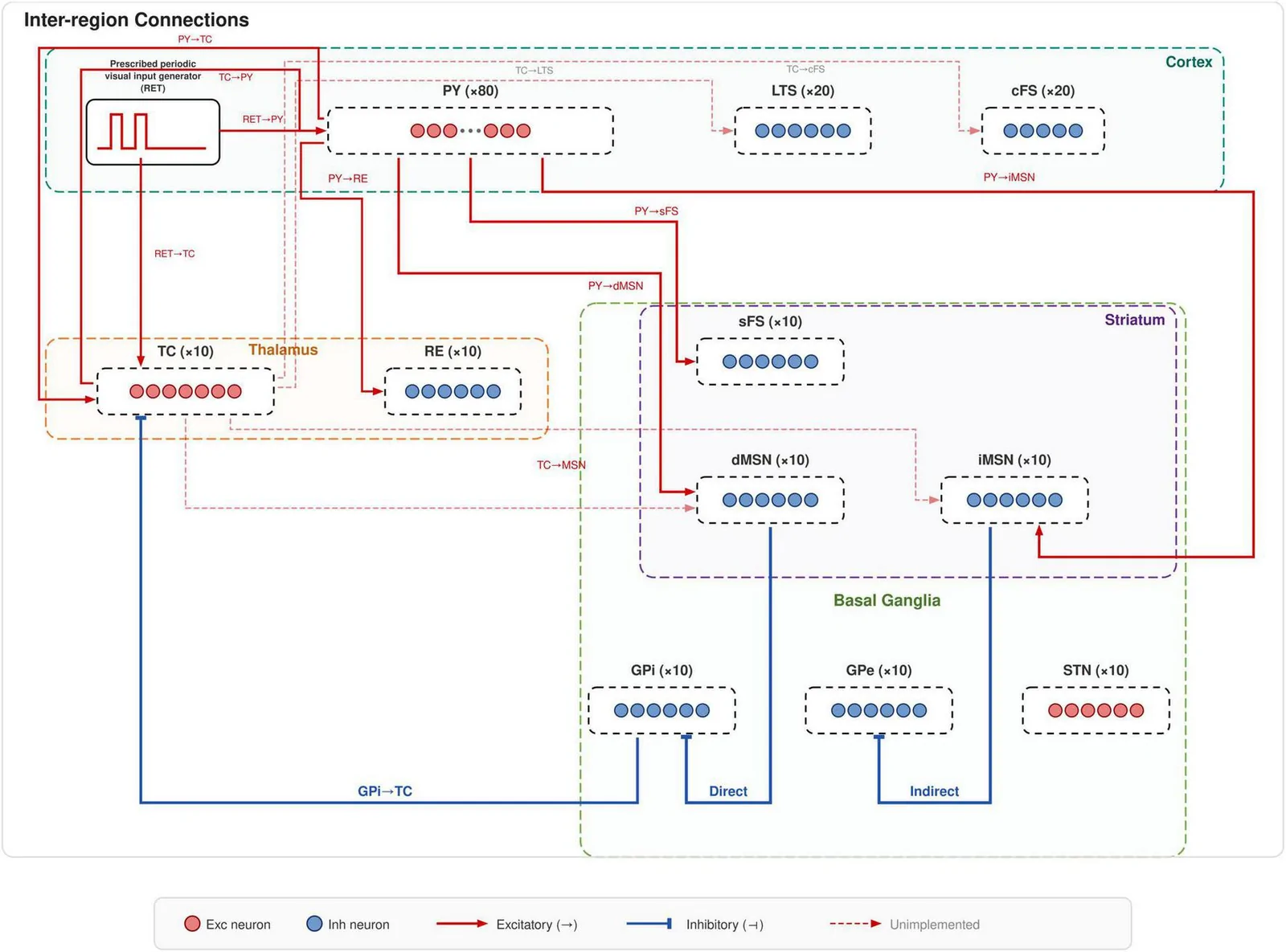
Inter-region connectivity of the CBT model. Excitatory projections are shown in red and inhibitory projections in blue; dashed pathways are anatomically recognized but not implemented. RET is a prescribed periodic functional visual input delivered to PY and TC. It is not an explicit retinal or LGN population; therefore, LGN filtering and adaptation are outside the model. Table 1 and Supplementary Figures 2, 3 provide population sizes and complete within-region connectivity.


$$\frac{C_{m}\,d\,V}{d\,t}=I_{a\,p\,p}-I_{l\,e\,a\,k}-I_{i\,o\,n}-I_{s\,y\,n}-I_{g\,a\,p}$$ (1)

In Equation 1, C_m_ denotes the specific membrane capacitance, I_ion denotes the sum of the voltage-gated ionic currents specific to each neuron type (e.g., I_Na, I_K, and the calcium and adaptation currents detailed in Supplementary Table 1), I_Leak represents the leakage current responsible for maintaining the resting potential, I_syn corresponds to chemical synaptic currents, and I_app indicates the externally applied injection current. Notably, the model incorporates a gap-junction current (I_gap) to simulate electrical synaptic coupling between striatal fast-spiking interneurons. This component is critical for forming local neural synchronization networks. For notational compactness, in the sFS sub-model of Supplementary Table 1, this gap-junction coupling is implemented within the I_app term as a coupling current proportional to the membrane-potential difference between connected interneurons.

#### 2.1.2 Ion channel dynamics

The model incorporates voltage-gated ion channels tailored to the electrophysiological behavior represented for each neuronal population. Fast sodium and delayed-rectifier potassium currents support action-potential generation. In contrast, T-type and L-type calcium currents, M-type potassium currents, and calcium-dependent afterhyperpolarization currents shape rhythmic and bursting behavior. Gating variables (x) follow the first-order kinetics given in Equation 2:


d⁢xd⁢t=(x∞(V)-x)τx(V) (2)

Here, in Equation 2, the steady-state activation/inactivation function x_∞(V)_ and time constant τ_*x*(V)_ are fitted using the Boltzmann distribution function. This precise mathematical formulation enables the model to reproduce complex neuronal discharge patterns. For instance, within the subthalamic nucleus and thalamic relay cells, a high density of I_CaT channels precipitates post-inhibitory rebound bursting during the depolarization phase (Ching et al., 2010; Mizrahi-Kliger et al., 2020). In the present model, this mechanism contributes to the PD-state circuitry by converting inhibitory inputs from the globus pallidus (GPe/GPi) into aberrant synchronized oscillatory outputs. Table 1 provides the detailed synaptic conductance, connection probabilities, and specific parameters governing interactions among these 11 populations and the retinal input unit.

**TABLE 1:** Synaptic connectivity matrix of the CBT network.

	Postsynaptic population										
Presynaptic population	PY	LTS	cFS	TC	RE	sFS	dMSN	iMSN	GPe	GPi	STN
PY		40 (0.01)	40 (0.04)	40 × 2 (0.0125)	40 × 2 (0.001)	4 × 2 (0.399)	4 × 2 (0.08)	4 × 2 (0.08)			
LTS	10 (0.032)	10 (0.015)									
cFS	10 (0.032)		10 (0.01)								
TC	10 (0.01)	10 (0.01)	10 (0.04)		10 (0.001)		3 (0.056)	3 (0.056)			
RE				10 (0.003)	9 (0.0067)						
sFS						2 (0.1)	3 (0.08)	3 (0.11)			
dMSN							2 (0.02)	2 (0.05)		3 (0.1)	
iMSN							3 (0.03)	2 (0.05)	4 (0.1)		
GPe									2 (0.5)	2 (0.5)	2 (0.5)
GPi				1 (0.17)							
STN									2 (0.15)	2 (0.15)	
RET	1 (0.01125)			1 (1.5)							

Entries give the number of presynaptic connections per neuron, with the per-neuron maximum synaptic conductance (nS) in parentheses unless otherwise specified. Rows indicate presynaptic neuron type and columns indicate postsynaptic neuron type. For GPi → TC only, 0.17 denotes the pathway-specific model-internal inhibitory coupling coefficient used in the TC synaptic-current implementation (implementation units), not a generic synaptic conductance in nS.

### 2.2 Pathological and physiological state emulation

The model defines two configurable state spaces: Parkinsonian progression and state-dependent wake, non-rapid-eye-movement (NREM) sleep, and anesthesia parameterizations.

#### 2.2.1 Parkinsonian pathology modeling (PD state)

Parkinson’s disease was represented as a dopamine-depletion-associated circuit state by modifying synaptic weights and background currents according to the component-model literature (Zhai et al., 2019; Mizrahi-Kliger et al., 2020). Cortical input to the direct pathway was reduced, input to the indirect pathway was increased, and sFS-mediated feedforward inhibition of MSNs was reduced. Background currents to GPe, GPi, and STN were also adjusted; for STN, the baseline current was reduced from 33 μA in the healthy configuration to 23 μA in the PD configuration. The resulting calibrated regime generated beta-range reorganization in selected basal-ganglia outputs. These outputs are treated as model-state readouts rather than as direct measures of therapeutic efficacy, and the parameterization is not claimed to reproduce every stage-specific PD phenotype.

#### 2.2.2 Consciousness and anesthesia states

State-dependent parameter sets were implemented for wakefulness, NREM sleep, and propofol anesthesia. NREM was represented by a low-frequency square-wave drive (2 Hz in baseline and 3 Hz in PD) and state-related leak-channel settings. Propofol was represented as a fourfold increase in maximal GABA_A conductance and decay time constant relative to wakefulness, targeting the modeled GABAergic projections (Table 2; Ching et al., 2010; Yoshida et al., 2018). These parameterizations allow cross-state simulations within the same architecture. In the present study, the propofol condition is used as a directional cross-state check against the increased subthalamic low-frequency power reported by Jiang et al. (2021). In contrast, the direct DBS-versus-visual comparison is restricted to the awake PD state. The simplified NREM setting does not reproduce all reported PD sleep phenomena and is treated as a limitation rather than a validated generalization.

**TABLE 2:** State-related parameters for physiological, pathological, and consciousness states.

Mechanism	Parameter	Baseline (wake)	PD (wake)	Modulation (NREM/anesthesia)
NREM slow-wave drive	Injection current duty cycle and frequency	Static	Static	NREM: 50% square wave, 2 Hz (baseline)/3 Hz (PD)
Anesthesia (propofol)	GABAR maximal conductance factor	×1	×1	×4 under anesthesia (RE → TC, cFS → PY, LTS → PY)
	GABA_A decay time constant (τ) factor	×1	×1	×4 under anesthesia
PD circuitopathy	Basal ganglia injection current I_app (μA)	GPe 21/GPi 22/STN 33	GPe 8/GPi 16/STN 23	–
	Striatum synaptic maximal conductance factor	×1	PY → sFS × 0.7; PY/TC → dMSN/iMSN × 0.85–0.95/×1.05–1.15 (weak/med/strong); sFS → dMSN/iMSN × 0.8	–

Dashes indicate parameters that are not modulated in the corresponding state.

### 2.3 Intervention protocols and quantitative metrics

The intervention protocols and outcome measures were defined to compare modeled network responses to DBS and prescribed visual drive without treating either simulation as evidence of clinical efficacy.

#### 2.3.1 Neuromodulation paradigms

Deep brain stimulation. STN-DBS was implemented as a symmetric, charge-balanced biphasic pulse train at 130 Hz with a pulse width of 0.06 ms per phase. The primary comparison used a model-internal current-density amplitude of 5 mA/cm^2^ (5000 μA/cm^2^), which should not be interpreted as a clinical device current. This strong-stimulation operating point was complemented by an amplitude analysis from 0 to 5 mA/cm^2^ at the same frequency and pulse width. The waveform follows the implementation used in the laboratory’s prior basal ganglia-thalamic (BGT)/DBS modeling framework (Cho et al., 2024).

Periodic visual input. The prescribed visual-input unit RET generated a prescribed 10 Hz base-pulse train and delivered it to PY and TC populations. Rhythmic-input effects have been studied computationally (Sasi and Sen Bhattacharya, 2022), but RET itself is not a biophysical retinal or LGN population; it represents an idealized prescribed periodic drive downstream of unmodeled retinal and LGN transformations. The omission of an explicit LGN is deliberate and keeps the study focused on how a temporally defined input perturbs the CBT network. Consequently, the model does not represent LGN filtering, contrast gain, adaptation, or jitter, and the magnitude of the simulated TC and cortical responses is conditional on the idealized temporal fidelity of RET. The modeled cross-modal influence reaches the motor-related network polysynaptically through the CBT architecture rather than through a claimed monosynaptic retinal projection (Foster et al., 2021; Gallivan and Goodale, 2018). The archived data key PD_visual40 is a legacy identifier and does not indicate a 40 Hz generator.

#### 2.3.2 Performance evaluation metrics

Spectral readouts. We computed power spectral density (PSD) from population-specific postsynaptic-current proxies rather than from extracellular local field potentials (LFPs) or raw membrane voltage. STN PSD specifically represents the population-summed outgoing AMPA current from STN to GPe and GPi. Absolute beta power was integrated over 13–30 Hz; cortical narrowband 38–42 Hz power was quantified as PY power over 38–42 Hz. The 38–42 Hz band is treated solely as an output spectral measure and not as the stimulation frequency or evidence of phase entrainment. The Beta Suppression Ratio (BSR) is reported descriptively relative to untreated PD, while inference is performed on run-level raw band power.

Population-output readouts. TC, RE, and cortical PY firing rates were calculated from rising-phase membrane-potential crossings through 0 mV. TC and RE PSD values are downstream synaptic-current proxies; a zero spectral proxy therefore indicates absent effective spiking-mediated output under that condition, not absent membrane dynamics. One full-resolution PD+DBS diagnostic inspected all 10 modeled TC cells over the 2,500 ms analyzed interval (250,000 native samples per cell). The local GPi → TC sensitivity analysis additionally retained voltage from all 10 TC cells in *N* = 10 matched-seed runs per scale at 0.8, 1.0, and 1.2 × nominal coupling; for storage and diagnostic analysis, the 500–3,000 ms interval was decimated to 1 kHz (2,500 samples per cell per run). The present CBT simulation does not include the controlled sensorimotor pulses used to quantify evoked relay error in related BGT models (Rubin and Terman, 2004; Guo et al., 2008; So et al., 2012; Cho et al., 2024). Accordingly, spontaneous TC firing is described as a population-output readout and is not interpreted as a direct measure of evoked relay fidelity. Time-aligned TC and PY membrane-potential or spike-train series were not jointly retained in the analysis archive used for this revision; retrospective thalamocortical coherence or phase-locking analysis was therefore not possible from the retained outputs.

### 2.4 Numerical implementation

#### 2.4.1 Solver and resolution

The conductance-based model was implemented in Python 3.9 using modular neuron, connectivity, and stimulation components. Hodgkin-Huxley ordinary differential equations were integrated with a fully implicit backward-Euler scheme, and the nonlinear algebraic system at each step was solved by Newton-Raphson iteration. The time step was 0.01 ms, and each run lasted 3,000 ms. The first 500 ms were prespecified as a transient and excluded to reduce the influence of initial conditions; this exclusion does not imply that initialization effects were eliminated. The modular implementation permits alternative cell, state, and stimulation components to be introduced without modifying the integration core.

#### 2.4.2 Latency simulation (shifting and stacking)

The original striatal component model used static currents to maintain baseline cFS and MSN activity (Yu and Wang, 2019), whereas the integrated CBT model supplies discrete PY and TC synaptic events. To reconcile these input representations, we used a signal-compatibility procedure termed shifting and stacking. We uniformly phase-shifted copies of the upstream pulse trains and summed them to generate a quasi-static mean drive while retaining time-varying fluctuations. This procedure is an engineering approximation for coupling the component models; it should not be interpreted as a biophysical model of axonal conduction delays. Supplementary Figure 6 shows its numerical behavior.

#### 2.4.3 Statistical analysis

For each primary condition, the network was simulated with *N* = 10 independent runs using different stochastic background realizations. Absolute band power was integrated from the raw, unnormalized PSD. We defined the BSR as (1 − P_treated/P_PD) × 100% from group means; it is therefore descriptive, and all inference was performed on run-level raw values. We analyzed PY firing, PY beta power (13–30 Hz), broadband gamma power (30–80 Hz), and narrowband 38–42 Hz power to provide cortical outputs. Planned pairwise comparisons used Welch’s two-sample *t*-test with Holm correction within each population and metric; Cohen’s d was reported as the standardized effect size. Deterministic zero-variance contrasts were evaluated using exact two-sided Mann–Whitney U tests; their unadjusted exact *p*-values and rank-biserial correlations are reported separately from the Holm–Bonferroni-corrected Welch tests. DBS-amplitude dependence was evaluated in a dedicated series at 0, 1, 2, 3, 4, and 5 mA/cm^2^ at 130 Hz and 0.06 ms per phase, with *N* = 10 independently generated runs per amplitude. The groups were not seed-matched; this is therefore an unpaired, between-run descriptive comparison rather than a paired within-run dose-escalation experiment. Dose results are reported as mean ± SD and as descriptive change relative to the 0 mA/cm^2^ group. No equivalence test was performed across amplitudes.

A second targeted, one-factor-at-a-time local sensitivity analysis varied the model-internal GPi → TC inhibitory coupling coefficient to 0.8, 1.0, and 1.2 times its nominal value of 0.17 (effective coefficients 0.136, 0.170, and 0.204) at the primary PD+DBS operating point (130 Hz, 5 mA/cm^2^, 0.06 ms per phase). Each scale used the same 10 stochastic seeds (2026081800–2026081809), giving 30 runs. Prespecified outputs were spontaneous TC firing, run-level TC voltage summaries and 0-mV crossings, and raw STN beta power integrated over 13–30 Hz. The run was the statistical unit; cell- and sample-level values were used only for voltage-distribution diagnostics. This analysis evaluates local robustness to one pathway coefficient at the selected PD+DBS point. It is not a global, multivariable, or interaction sensitivity analysis. Full numeric summaries and provenance are provided in Supplementary Tables 2–7.

A third targeted, matched-seed one-factor-at-a-time local sensitivity analysis varied GABA_A decay time constant, STN–GPe core-loop coupling conductance, and STN injected current by ±20%. Each parameter-condition cell used the same five stochastic seeds (2026082400–2026082404). M1–M2 were evaluated under PD+DBS and M3–M4 under PD+periodic visual input; M1 and M3 used a single shared nominal PD denominator rather than a separately rerun untreated baseline at every parameter level. This targeted local analysis did not test combined-parameter interactions or global robustness (Supplementary Table 7).

## 3 Results

We first characterize the calibrated CBT model and then compare the spectral and population-output responses produced by DBS and prescribe periodic visual input implemented with 10 Hz base pulses. All interpretations are restricted to the modeled signals and parameter regime.

### 3.1 Model characterization and evidence checks

We report three complementary evidence levels: reproduction of selected reference behaviors used to characterize the calibrated regime, a directional external comparison under propofol, and numerical/run-to-run behavior across stochastic realizations. These checks do not establish performance in untested conditions or global robustness to structural parameters. Unless otherwise stated, values were calculated from *N* = 10 independent 3,000 ms simulations after excluding the first 500 ms, using a 0.01 ms implicit time step. PSD curves show raw, unnormalized power from the population-specific synaptic-current proxies defined in Section “2.3.2 Performance evaluation metrics,” and firing rates were calculated from rising-phase 0-mV crossings.

#### 3.1.1 Reproduction of selected reference behaviors

We used three outputs to characterize the calibrated healthy and PD regimes. First, healthy-state sFS, dMSN, and iMSN firing rates were 5.53 ± 1.13, 2.27 ± 0.28, and 1.83 ± 0.32 Hz, respectively, consistent with the low-rate striatal regime of Yu and Wang (2019). Second, dopamine depletion increased raw beta power in GPi by 3.8-fold and in iMSN by 4.0-fold relative to healthy (Holm-corrected Welch *p* < 10^–9^; *N* = 10), although the STN current proxy did not show the canonical clinical STN local-field-potential (LFP) increase (Section “4.3.4 Directionality of the subthalamic beta proxy”). Third, dMSN firing increased from 2.3 to 5.4 Hz and iMSN firing from 1.8 to 9.1 Hz (both Holm-corrected *p* < 10^–9^). These comparisons document selected behaviors of the calibrated implementation; they do not validate every biological feature of PD.

#### 3.1.2 Directional cross-state check: propofol-associated low-frequency enhancement

The propofol parameter set was specified from GABAergic synaptic pharmacology rather than from an STN spectral target. In the PD network, simulated propofol increased raw STN delta/theta power (1–8 Hz) from 1.25 ± 0.30 × 10^–3^ a.u. in wakefulness to 68.2 ± 31.9 × 10^–3^ a.u., a 54-fold change (Holm-corrected Welch *p* < 10^–9^; *N* = 10). The direction is consistent with the increased low-frequency STN activity reported intraoperatively by Jiang et al. (2021). This comparison supports cross-state use of the architecture but does not establish that intervention effects observed in wakefulness persist under anesthesia.

#### 3.1.3 Numerical behavior and run-to-run dispersion

The integrated simulation remained numerically finite over 3,000 ms at the 0.01 ms implicit time step for the analyzed runs. The code-audited RET generator produced 1 ms pulses at 100 ms intervals (10 Hz; Supplementary Figure 1). Across *N* = 10 stochastic realizations, the coefficient of variation of raw STN beta power was 32.8% in PD, 21.1% under DBS, and 19.4% under periodic visual input; the coefficient of variation of TC firing was ≤4% in conditions with nonzero firing. These values characterize numerical behavior and run-to-run dispersion for the tested configurations only; they do not indicate robustness to untested conductances, connection weights, delays, frequencies, or waveforms.

### 3.2 Detailed reproduction of pathological beta oscillations

We examined the reorganization produced by the calibrated dopamine-depletion parameter set. In Figure 2A, beta-band (13–30 Hz) power increased in GPi and indirect-pathway iMSN as PD severity progressed. In contrast, the model-internal outgoing STN current proxy did not show the canonical clinical STN-LFP increase (Section “4.3.4 Directionality of the subthalamic beta proxy”). Striatal firing also shifted: dMSN firing increased from 2.3 to 5.4 Hz and iMSN firing from 1.8 to 9.1 Hz (Figure 2B). These results are selected benchmark checks for pallidal, striatal, and spectral outputs and do not validate every biological feature of PD.

**FIGURE 2:**
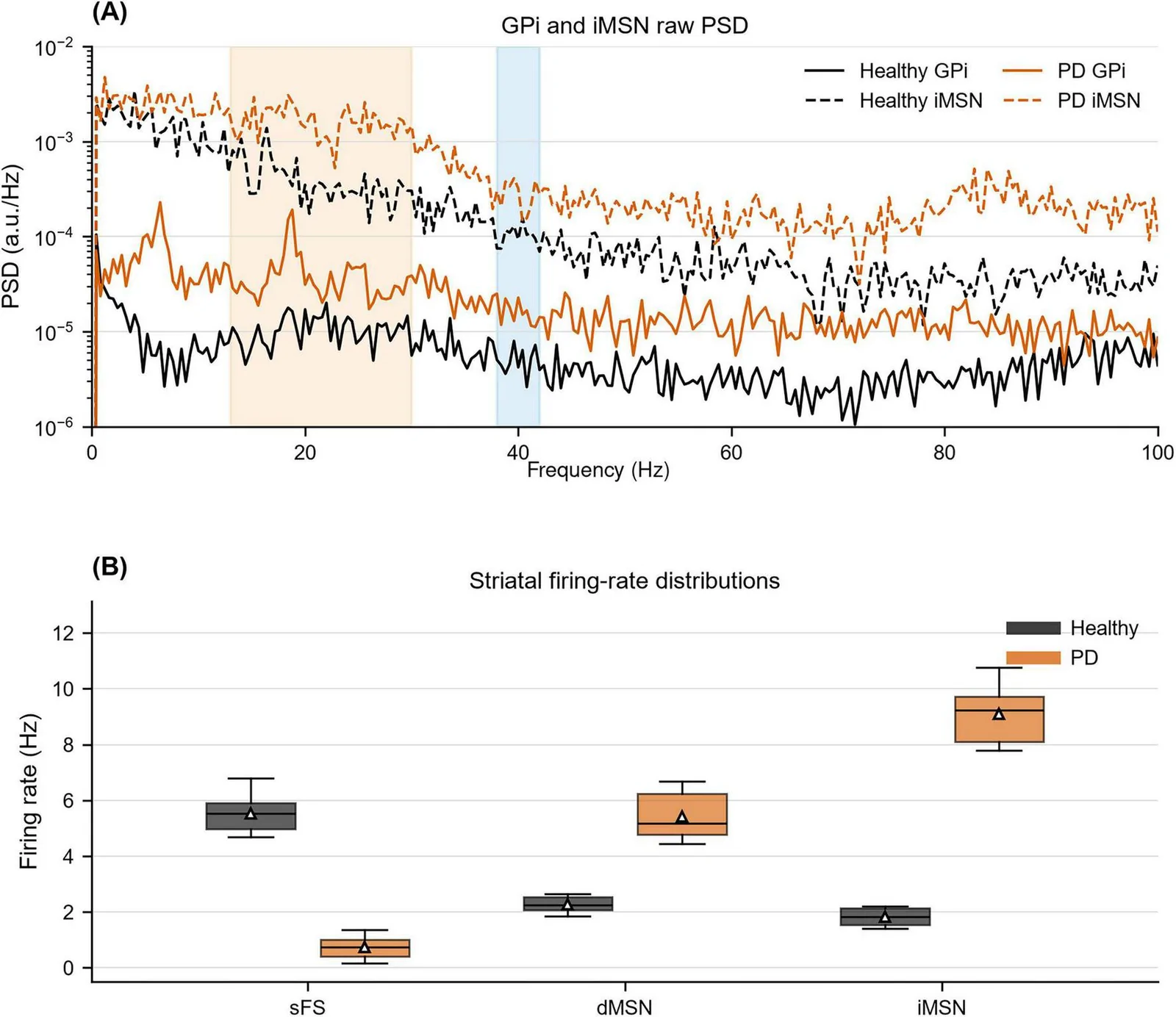
Selected reference behaviors of the calibrated CBT model. **(A)** Raw PSD of beta-range activity in Healthy and PD conditions, plotted on a logarithmic ordinate for GPi (solid lines) and indirect-pathway iMSN (dashed lines); shaded bands mark 13–30 Hz and 38–42 Hz. **(B)** Striatal firing-rate distributions across *N* = 10 runs for sFS, dMSN, and iMSN populations; gray and orange boxes represent Healthy and PD runs, respectively. Boxes indicate the interquartile range and median, whiskers the non-outlier range, and white triangles the group mean. These panels characterize selected outputs and do not validate every biological feature of PD.

### 3.3 Numerical characterization of the coupling procedure

Supplementary Figure 6 characterizes the shifting-and-stacking procedure used to couple dynamic PY/TC events to the striatal component model. The time-averaged synthesized input approximated the reference static drive while retaining time-varying fluctuations. This check supports numerical compatibility of the coupled implementation for the tested configuration; it does not establish that the transformation reproduces biological axonal-delay statistics or synaptic integration *in vivo*.

### 3.4 Comparative model responses to DBS and periodic visual input

We compared both perturbations on the same awake PD network. DBS was evaluated primarily against the model-internal STN beta measure, whereas visual input was evaluated against thalamic and cortical population outputs. These are complementary readouts, not interchangeable measures of clinical efficacy (Figure 3).

**FIGURE 3:**
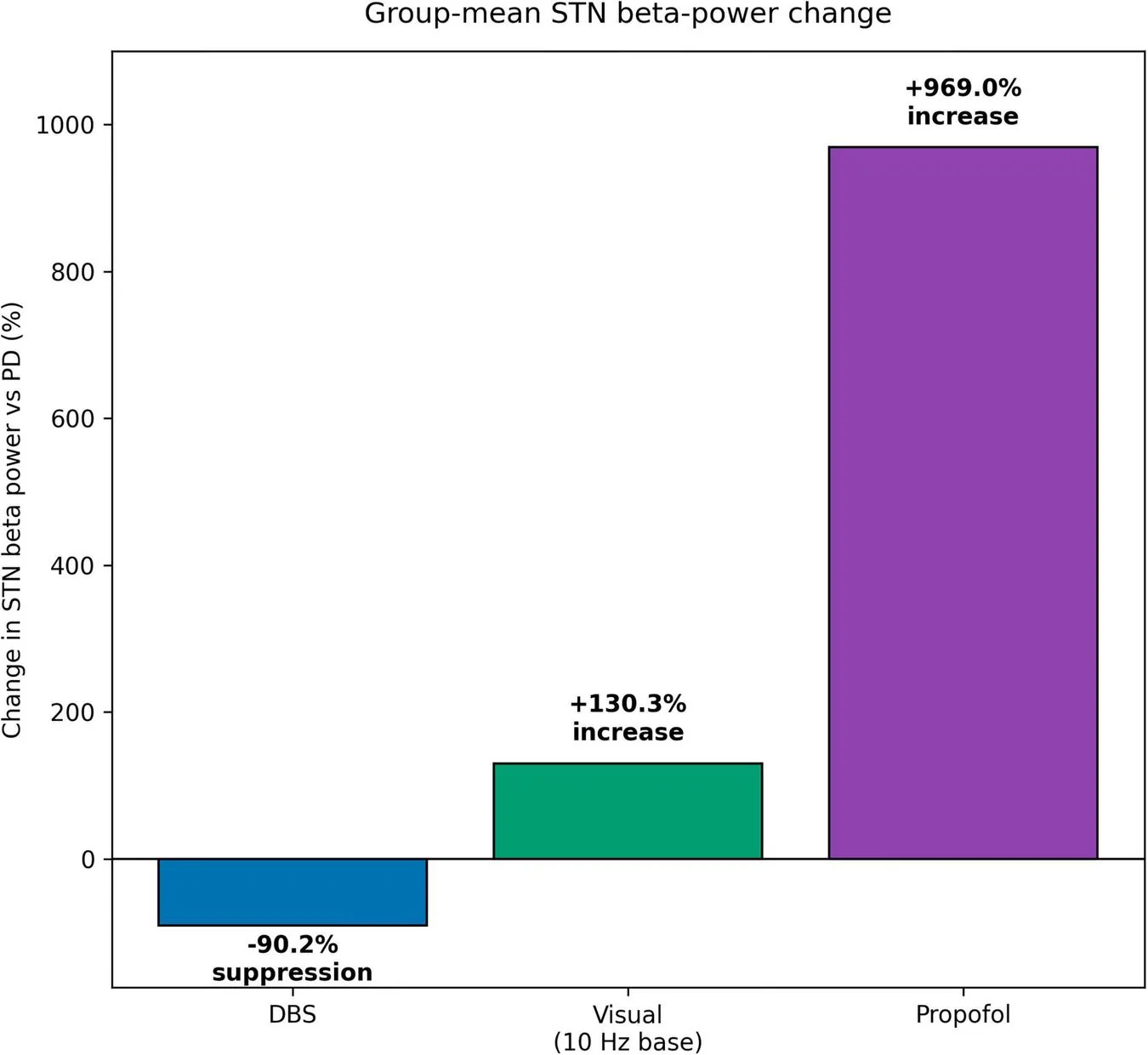
Group-mean change in raw STN beta power relative to PD (*N* = 10). The STN measure is a model-internal proxy for outgoing synaptic current. DBS decreases this measure, whereas periodic visual input and propofol increase it through different modeled spectral regimes. Values are descriptive ratios of group means; inference is performed on run-level raw beta power.

At the primary 5 mA/cm^2^ operating point, 130 Hz STN-DBS reduced raw STN beta power from 0.0184 ± 0.0060 a.u. in PD to 0.0018 ± 0.0004 a.u. (Holm-corrected Welch *p* = 1.1 × 10^–5^; Cohen’s *d* = 3.9; *N* = 10; Figure 4A), corresponding to a descriptive BSR of 90.2%. STN threshold crossings occurred at the stimulation frequency and are therefore treated as stimulation-locked events rather than spontaneous firing. TC spontaneous firing decreased from 8.6 ± 0.3 Hz in PD to 0 Hz under this DBS configuration (Figure 4B). The Healthy condition also yielded 0 Hz in this spontaneous readout; the shared zero value does not establish physiological equivalence or evoked relay competence, neither of which was tested. This output is described as spontaneous TC quiescence; by itself, it does not establish a reversible lesion, depolarization block, or failure of evoked sensorimotor relay.

**FIGURE 4:**
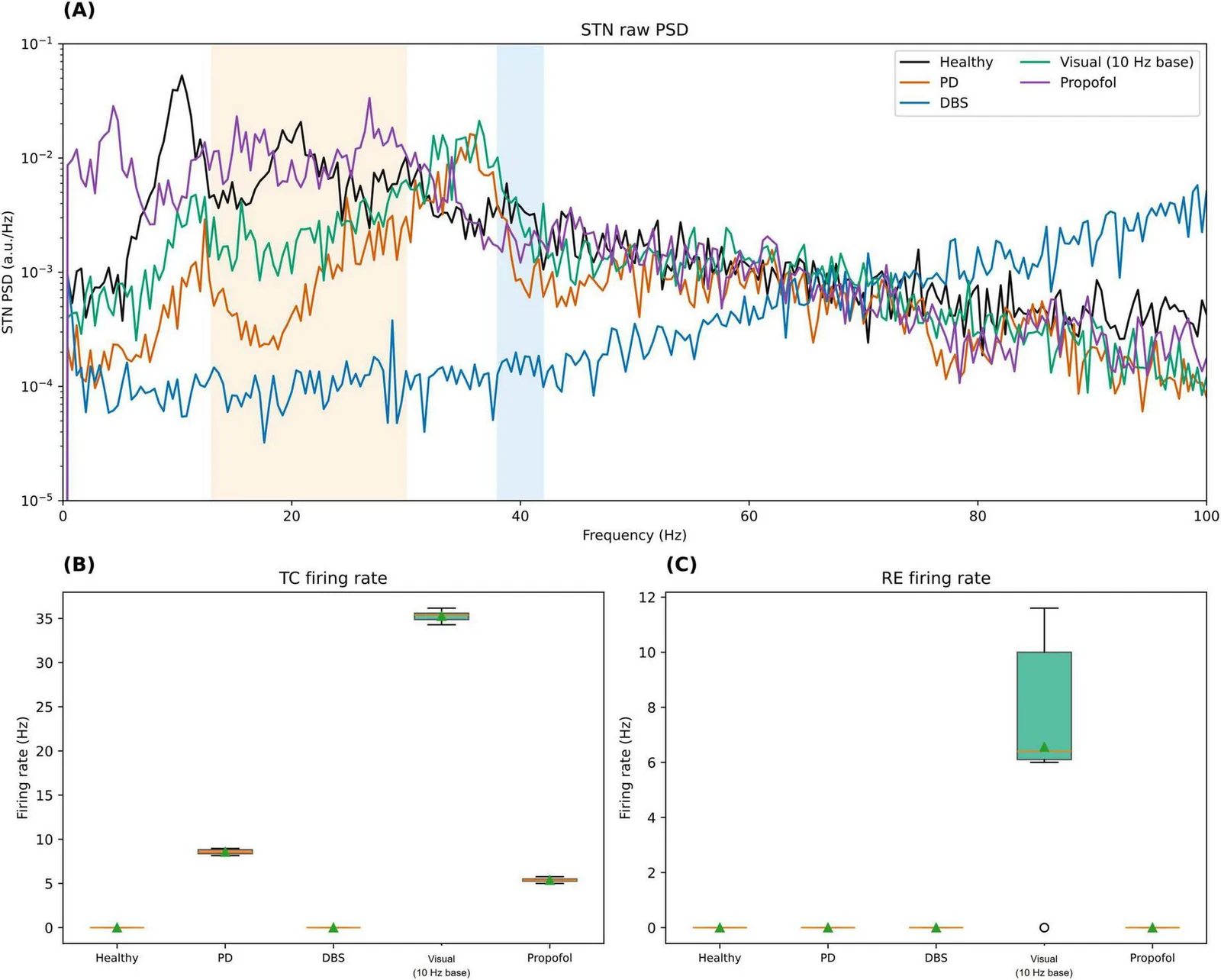
Network outputs across Healthy, PD, PD+DBS, PD+periodic visual-input, and PD+propofol conditions (*N* = 10 per condition). **(A)** Raw STN synaptic-current proxy PSD. **(B)** TC firing distributions: spontaneous TC firing is 0 Hz in Healthy and under the selected DBS condition and increased under periodic visual input; the shared zero value does not establish physiological equivalence or evoked relay competence. This panel does not test evoked relay fidelity. **(C)** RE firing distributions, showing recruitment under periodic visual input. Boxes show interquartile ranges and medians, whiskers the non-outlier range, and white triangles the mean.

Periodic visual input did not suppress the STN beta proxy: raw STN beta power was 0.0423 ± 0.0082 a.u., an increase relative to PD (Holm-corrected *p* = 2.3 × 10^–6^). Instead, the input increased TC firing from 8.6 ± 0.3 to 35.3 ± 0.6 Hz and recruited RE firing from 0 to 6.56 ± 4.15 Hz (Figures 4B,C). These findings indicate that the prescribed rhythm produces a different modeled response profile from DBS–greater thalamic activity and increased 38–42 Hz output-band power without STN-beta suppression–rather than demonstrating a superior or clinically activity-preserving treatment mechanism. Table 3 summarizes the principal prespecified statistical contrasts.

**TABLE 3:** Statistical summary (*N* = 10 per condition).

Contrast	Metric	Value	*P*-value	Effect size
PD vs. Healthy	GPi beta power (fold)	3.8×	<10^–9^	–
PD vs. Healthy	iMSN beta power (fold)	4.0×	<10^–9^	–
PD vs. Healthy	dMSN firing (Hz)	2.3 → 5.4	<10^–9^	–
PD vs. Healthy	iMSN firing (Hz)	1.8 → 9.1	<10^–9^	–
PD+DBS vs. PD	STN beta power (a.u.)	0.0184 → 0.0018	1.1 × 10^–5^	3.9
PD+DBS vs. PD	BSR (%)	90.2	–	–
PD+DBS vs. PD	TC firing (Hz)	8.6 → 0	1.08 × 10^–5^	Rank-biserial *r* = 1.0
PD+periodic visual input vs. PD	STN beta power (a.u.)	0.0184 → 0.0423	2.3 × 10^–6^	–
PD+periodic visual input vs. PD	TC firing (Hz)	8.6 → 35.3	<10^–8^	–
PD+periodic visual input vs. PD	RE firing (Hz)	0 → 6.56	1.5 × 10^–3^	Rank-biserial *r* = 1.0
Propofol vs. PD wake	STN delta+theta (a.u.)	1.25 × 10^–3^ → 68.2 × 10^–3^	<10^–9^	>9
PD vs. Healthy	PY firing (Hz)	5.83 → 4.86	0.016	*d* = −1.36
DBS vs. PD	PY firing (Hz)	4.86 → 5.28	0.285	*d* = 0.49
Periodic visual vs. PD	PY firing (Hz)	4.86 → 10.33	1.18 × 10^–10^	*d* = 6.47
Periodic visual vs. PD	PY 38–42 Hz power	0.125 → 0.514	7.90 × 10^–5^	*d* = 3.26

Arrows indicate group means; fold changes are ratios of group means. The BSR is a derived descriptive quantity computed from the ratio of group mean powers and therefore carries no standard deviation of its own; inferential statistics for that contrast are those of the underlying raw beta-power comparison listed immediately above, and are not repeated on the BSR row. Welch’s two-sample tests were Holm–Bonferroni corrected within each prespecified population-metric family. Deterministic zero-variance contrasts were evaluated using exact two-sided Mann–Whitney U tests and are reported as unadjusted exact *p*-values with rank-biserial correlations. BSR is computed from raw (un-normalized) beta power. Dashes indicate values not applicable or not separately reported.

### 3.5 Cortical outputs and targeted sensitivity analyses

Cortical PY outputs differed across the four principal conditions (Figure 5 and Supplementary Table 2). Mean firing was 5.83 ± 0.56 Hz in Healthy, 4.86 ± 0.84 Hz in PD, 5.28 ± 0.87 Hz under DBS, and 10.33 ± 0.85 Hz under periodic visual input. PD was lower than Healthy (Holm-corrected *p* = 0.016; *d* = −1.36). Relative to PD, DBS did not significantly change PY firing (*p* = 0.285; *d* = 0.49), beta power (*p* = 0.583), gamma power over 30–80 Hz (*p* = 0.798), or 38–42 Hz power (*p* = 1.000). Periodic visual input increased PY firing (*p* = 1.18 × 10^–10^; *d* = 6.47), beta power (*p* = 6.26 × 10^–6^), gamma power over 30–80 Hz (*p* = 6.29 × 10^–6^), and 38–42 Hz power (*p* = 7.90 × 10^–5^; Figures 5A–D). Thus, the cortical result supports input-driven recruitment and broad output-band spectral recruitment, not cortical beta normalization, phase entrainment, or preservation of cortical processing.

**FIGURE 5:**
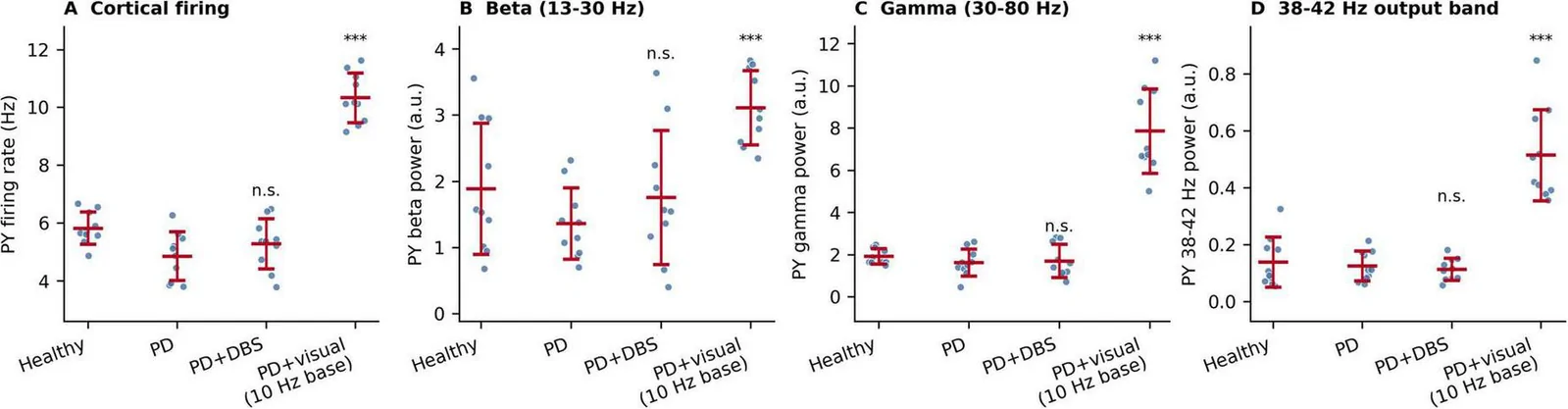
Cortical PY outputs across Healthy, PD, PD+DBS, and PD+periodic visual-input conditions (*N* = 10 independent runs per condition). **(A)** Firing rate. **(B)** Raw beta power (13–30 Hz). **(C)** Raw gamma power (30–80 Hz). **(D)** Raw narrowband power (38–42 Hz). Blue points denote individual stochastic runs; red bars denote mean ± SD. Significance labels compare DBS or periodic visual input with PD using Welch’s two-sample *t*-test with Holm correction within each metric (n.s., not significant; ***p < 0.001). Full statistics are provided in Supplementary Table 2.

The dedicated, unpaired amplitude comparison showed a non-monotonic response rather than a linear dose relationship (Supplementary Figure 4 and Supplementary Table 3). At 0 mA/cm^2^, the STN current-proxy beta measure was 0.0268 ± 0.0158 a.u. and TC spontaneous firing was 8.27 ± 0.45 Hz. At 1 mA/cm^2^, the STN beta measure increased to 0.0529 ± 0.0075 a.u., while TC spontaneous firing fell to 0 Hz. For every nonzero level from 1 to 5 mA/cm^2^, TC spontaneous firing was 0 ± 0 Hz across *N* = 10 runs per level (50 stimulated run-level outputs in total). At 2, 3, 4, and 5 mA/cm^2^, mean STN beta values occupied a narrow range of 0.00186–0.00212 a.u., corresponding to 92.1%–93.1% descriptive suppression relative to the 0 mA/cm^2^ group; mean PY firing ranged from 5.65 to 6.11 Hz. The dedicated 5 mA/cm^2^ mean (0.00195 ± 0.00041 a.u.) was numerically close to the primary 5 mA/cm^2^ result (0.00181 ± 0.00038 a.u.), but no formal equivalence test was performed. The primary comparison, dedicated dose series, and three-parameter local sensitivity analysis used separate stochastic ensembles and respective PD or 0 mA baselines; their descriptive suppression percentages (90.2%, 92.1%–93.1%, and nominal 94.2%) therefore should not be treated as estimates from a common denominator. These findings show that the original 5 mA/cm^2^ observation is not isolated within the tested amplitude series; they do not establish global parameter robustness.

To characterize the zero TC firing value, we inspected modeled afferents and membrane voltage. In one full-resolution PD+DBS diagnostic containing all 10 TC cells, all 2,500,000 analyzed voltage samples were finite; the population-wide range was −71.10 to −57.92 mV, and no sample crossed 0 mV. PY → TC excitation and GPi → TC inhibition were both nonzero, whereas RET was inactive because no visual drive was applied and RE output was silent in this resting DBS condition. In that run, the voltage distribution did not support sustained depolarization block and was most consistent with subthreshold spontaneous quiescence under the calibrated input balance. We then varied GPi → TC coupling locally to 0.8, 1.0, and 1.2 × nominal using *N* = 10 matched seeds per scale (Supplementary Figure 5 and Supplementary Tables 5, 6). TC spontaneous firing remained 0 Hz in 30/30 runs. Run-mean TC voltages were −67.227 ± 0.035, −67.903 ± 0.038, and −68.497 ± 0.039 mV at 0.8, 1.0, and 1.2 × nominal, respectively. All 750,000 saved samples were finite; the overall range was −72.058 to −54.742 mV, with no samples above −30 mV and no 0-mV crossings. Raw STN beta power was 0.001931 ± 0.000333 a.u. at each scale. With identical seeds, TC remaining silent at all three scales produced no scale-dependent feedback difference to the upstream STN signal, explaining the numerically identical STN-beta values rather than indicating duplicated observations. Thus, the zero-firing output was not confined to the nominal GPi → TC coefficient and remained consistent with subthreshold quiescence across the tested ±20% range. To probe the parameter classes highlighted in the review, we additionally ran a matched-seed (*N* = 5), one-factor-at-a-time ±20% local sensitivity analysis of a GABA_A decay-time-constant factor, the STN–GPe core-loop coupling coefficient, and STN injected current. M1 and M3 were calculated by trapezoidal integration of raw STN PSD over 13–30 Hz. All four principal outcomes were preserved across five finite runs per parameter-condition cell: DBS raw STN-beta suppression remained 88.9%–95.8% (nominal 94.2%), TC spontaneous firing under DBS remained 0 Hz in 35/35 runs, periodic visual input increased raw STN beta by 76.2%–230.9% relative to nominal PD, and TC firing under periodic visual input increased 3.70–4.29-fold (Supplementary Table 7). Two removable singularities in channel-rate expressions were evaluated with numerically stable analytic limits; only the two originally non-finite runs were rerun, using their original matched seeds, and all other model-loop outputs were retained unchanged. These local analyses do not exclude dependence on untested conductances, delays, waveforms, or parameter interactions, and no controlled sensorimotor probe was delivered to test evoked relay fidelity.

## 4 Discussion

This study presents a conductance-based CBT testbed that combines PD, arousal-state, and stimulation configurations within one architecture. The results should be read as model-generated response patterns and hypotheses, not as demonstrations of biological mechanisms or therapeutic efficacy.

### 4.1 Bridging the gap: a unified framework for pathology and consciousness

The same CBT architecture contains parameterizations for wakefulness, NREM sleep, and propofol anesthesia. In this study, the propofol condition provides a directional cross-state check against the low-frequency STN enhancement reported by Jiang et al. (2021), while the head-to-head DBS-versus-visual comparison remains deliberately restricted to resting wakefulness. Propofol modifies GABA_A conductance and decay kinetics in the thalamocortical component (Ching et al., 2010). In contrast, NREM-related leak and background-drive changes alter the operating state in which sensory and basal-ganglia inputs are integrated. Parkinsonian beta activity can also persist during NREM sleep (Mizrahi-Kliger et al., 2020). Because these state variables directly shape STN spectra and thalamocortical responsiveness, the awake intervention contrast should not be assumed to remain quantitatively invariant during sedation or sleep; a future factorial comparison across intervention and arousal state is warranted.

The cross-state implementation provides evidence of configurability, not universal validation. In particular, the simplified NREM setting does not reproduce the sleep-related pathological beta reported clinically, and the propofol comparison addresses spectral direction rather than clinical electrode-placement performance or treatment response. These boundaries are now stated explicitly.

### 4.2 Model-generated contrast between DBS and visual input

The simulations suggest that DBS and prescribed periodic visual input affect different model outputs. This contrast is useful for forming experimentally testable hypotheses, but the present study cannot establish that the same causal mechanisms operate in the biological brain.

#### 4.2.1 DBS: beta suppression and state-dependent TC quiescence

High-frequency DBS reduced the model-internal STN beta proxy. TC spontaneous firing was 0 ± 0 Hz at every nonzero amplitude from 1 to 5 mA/cm^2^ (*N* = 10 per level). In the full-resolution all-cell diagnostic, 2.5 million finite voltage samples remained between −71.10 and −57.92 mV with no 0-mV crossing, while PY → TC and GPi → TC currents were nonzero. The matched-seed GPi → TC analysis extended this observation across *N* = 10 runs at each of 0.8, 1.0, and 1.2 × nominal coupling: TC firing remained 0 Hz in 30/30 runs, and 750,000 saved voltage samples remained subthreshold. Collectively, these diagnostics do not support sustained depolarization block in the examined simulations and instead support a model-conditional interpretation of subthreshold spontaneous quiescence across the tested local GPi → TC range. They reduce concern that the result arises only from the nominal GPi → TC coefficient, but they do not establish robustness to other connectivity parameters or parameter interactions. Related BGT models evaluate a different endpoint by applying explicit sensorimotor pulses and scoring evoked relay errors; those studies show that relay fidelity depends on the temporal pattern of pallidal inhibition and may improve during high-frequency DBS despite strong or tonic GPi output (Rubin and Terman, 2004; Guo et al., 2008; So et al., 2012; Cho et al., 2024). Because the present CBT simulation did not deliver that probe, TC = 0 is not interpreted as a reversible lesion, indiscriminate information blockade, or failed evoked relay.

#### 4.2.2 Visual input: thalamic and cortical recruitment

Periodic visual input increased TC, RE, and cortical PY activity and produced a clear increase in PY 38–42 Hz power. This is consistent with input-driven recruitment of the modeled thalamocortical populations. However, it did not normalize PY beta or suppress the STN beta proxy, and the idealized RET input bypasses explicit LGN filtering. The result therefore supports the narrower hypothesis that a temporally precise visual drive can recruit downstream activity in this calibrated network. Whether such recruitment preserves information processing, improves motor behavior, or offers clinical benefit requires evoked-task, animal, and human validation.

### 4.3 Critical limitations and future perspectives

The following limitations define the boundary conditions of the current evidence and the analyses required to test structural robustness and translational relevance.

#### 4.3.1 Targeted sensitivity and the calibrated parameter regime

We performed three targeted sensitivity analyses without changing the CBT architecture. First, the dedicated 0–5 mA/cm^2^ series addressed the intervention parameter most directly linked to the primary analysis. All six amplitude groups used the same architecture, duration, transient exclusion, stimulation frequency, pulse width, and analysis pipeline; the groups were independent and unpaired because the stochastic runs were not seed-matched. Mean STN beta values at 2–5 mA/cm^2^ occupied a narrow range, showing that the low value at 5 mA/cm^2^ was not isolated, while the 1 mA/cm^2^ increase showed a non-monotonic response. Because no amplitudes between 0 and 1 mA/cm^2^ were sampled, the transition to zero spontaneous TC firing within that interval remains unresolved. Second, a matched-seed one-factor-at-a-time analysis varied GPi → TC inhibitory coupling by ±20% at the primary PD+DBS point. TC firing remained 0 Hz in 30/30 runs, the saved TC voltage remained subthreshold, and raw STN beta was descriptively unchanged across the three scales. This directly evaluates one connectivity parameter implicated in the TC result, but all other conductances, currents, connection weights, delays, frequencies, and waveforms remained fixed. A prior basal ganglia-thalamic (BGT)/DBS implementation from our group examined 0–5000 μA/cm^2^ and 100–185 Hz as continuous DBS control domains (Cho et al., 2024), and independent conductance-based BGT models report frequency-, pattern-, and activation-dependent changes in thalamic relay (Rubin and Terman, 2004; Guo et al., 2008; So et al., 2012). These studies support parameter-range selection and cross-model context; they are not empirical sensitivity evidence for the present CBT architecture. Hopf-bifurcation studies likewise show that changes in coupling and delay can reorganize oscillatory states (Wang et al., 2022, 2023a,2023b). Third, a matched-seed ±20% one-factor-at-a-time analysis of a GABA_A decay factor, the STN–GPe coupling coefficient, and STN-injected current left all four principal outcomes qualitatively unchanged across five finite runs per parameter-condition cell. Raw STN-beta suppression under DBS remained 88.9%–95.8%, while periodic visual input increased raw STN beta by 76.2%–230.9% and TC firing by 3.70–4.29-fold relative to nominal PD (Supplementary Table 7). We nonetheless limit the conclusions to the tested local parameter domains and do not claim global, multivariable, or interaction robustness (Supplementary Tables 4, 7).

#### 4.3.2 Exaggerated cortical synchronization

We noticed that across all simulated states, cortical pyramidal cells exhibit stronger high-beta (20–30 Hz) synchronized oscillations than would be clinically expected. Although this does not alter the overall trend of PD pathology generation, it may stem from our simplifying assumptions regarding the cortical module. The current model focuses primarily on the vertical “cortico-basal ganglia-thalamic” loop while simplifying horizontal “intra-cortical” inhibitory connections. This simplification may lead to hyperexcitability in local cortical circuits. Future model iterations should introduce more complex microcircuitry, including interactions between interneurons across layers, to correct this spectral bias.

#### 4.3.3 The complexity of sleep micro-architecture

The simplified NREM implementation did not reproduce significant pathological beta oscillations in the combined PD+NREM condition, in contrast to evidence that parkinsonian beta activity can persist during NREM sleep (Mizrahi-Kliger et al., 2020). The current NREM state is represented primarily through duty-cycle input and selected leak-current settings and therefore omits broader neuromodulatory changes involving acetylcholine, serotonin, and potassium-channel regulation (Yoshida et al., 2018). Propofol, NREM, and wakefulness modify variables that also shape the response to GPi inhibition and periodic visual drive. The direction and magnitude of the DBS-versus-visual contrast therefore cannot be inferred from the awake simulations. Future work should incorporate the omitted state mechanisms and test the interventions factorially across wake, NREM, and anesthesia rather than extrapolating from the present comparison.

#### 4.3.4 Directionality of the subthalamic beta proxy

A canonical signature of PD in human local-field potential (LFP) recordings is an increase in subthalamic beta-band power; in our model, however, the outgoing STN → GPe/GPi AMPA-current proxy used to compute STN power spectral density decreases from the healthy to the PD wake state. In contrast, the canonical PD-related rise emerges in GPi and in indirect-pathway iMSNs instead (Section “3 Results”). Two factors plausibly contribute to this directionality. First, our STN PSD is derived from the outgoing STN → GPe/GPi AMPA-current proxy rather than from a simulated extracellular STN local-field potential; in the present network, the dominant pathological beta is generated and most strongly expressed in GPi and iMSNs, with STN inheriting it through synaptic projections, and the outgoing STN → GPe/GPi current proxy therefore captures only part of the pathological oscillation. Second, the model omits a direct hyper-direct cortico-subthalamic projection, which is now thought to be an important driver of STN beta in human PD (Herrington et al., 2016). As a consequence, in this study STN beta should be interpreted as a model-internal index of pathological STN drive rather than as a direct surrogate of human STN-LFP beta; quantitative comparisons across treatments are therefore reported as signed power changes relative to the model’s own PD baseline, and the canonical PD-related rise is documented in GPi/iMSN where the model does reproduce it. Adding an explicit LFP forward model and a hyper-direct pathway is a defined direction for future testbed revisions.

#### 4.3.5 Periodic visual input and the subthalamic spectrum

Under periodic visual input, raw STN beta power increased rather than decreased relative to untreated PD. The prescribed periodic input and network-generated components add spectral energy to the model-internal STN synaptic-current proxy. This observation is not evidence that visual stimulation reduces human STN-LFP beta. At the cortical level, visual input likewise increased both PY beta and 38–42 Hz power, indicating broad input-driven spectral recruitment rather than selective beta normalization. The appropriate conclusion is therefore that the visual input and DBS produce distinct modeled spectral and population-output profiles.

#### 4.3.6 LGN, evoked-relay, and translational scope

RET supplies an idealized periodic drive and bypasses explicit LGN dynamics. The model therefore cannot quantify how LGN filtering, temporal jitter, adaptation, or contrast gain would change the periodic-input response magnitude. Similarly, spontaneous TC firing does not measure evoked sensory-motor information transfer. Finally, model current density is not directly equivalent to clinical DBS-device current because it does not account for electrode geometry, volume conduction, axonal recruitment, or patient variability. The study consequently proposes experimental hypotheses and analysis targets rather than treatment recommendations.

## 5 Conclusion

We developed a multi-state, conductance-based CBT testbed. We used it to compare 130 Hz STN-DBS with a prescribed periodic visual input implemented with 10 Hz base pulses on the same awake PD network. At the examined operating points, DBS was associated with lower model-internal STN beta power, whereas periodic visual input increased thalamic and cortical activity without suppressing that measure. The TC = 0 output persisted across 1–5 mA/cm^2^ and across a matched-seed ±20% local variation in GPi → TC coupling; all saved TC voltages remained subthreshold, a pattern that did not support sustained depolarization block and was consistent with spontaneous quiescence in the tested simulations. Cortical analyses showed that visual input increased PY firing and 38–42 Hz power, while DBS did not significantly restore PY firing relative to PD. The matched-seed ±20% GABA_A-decay, STN–GPe-coupling, and STN-current analysis preserved all four prespecified outcomes within the tested local domains. These results define testable, model-conditional hypotheses about how the two perturbations redistribute network activity. They do not establish evoked relay competence, clinical efficacy, treatment superiority, global parameter robustness, or a universal neuromodulation design principle; those questions require broader multivariable sensitivity analyses and experimental validation.

## Data Availability

The original contributions presented in this study are included in this article/Supplementary material, further inquiries can be directed to the corresponding author.
