# Supplementary material for: An in silico cortico-basal ganglia-thalamic testbed for comparing visual and deep brain stimulation in Parkinson’s disease
Source: Front Comput Neurosci. 2026 Oct 2;20:1950516. doi: 10.3389/fncom.2026.1950516 (PMC13672486; doi:10.3389/fncom.2026.1950516)
Supplement: Supplementary file 1 [file Data_Sheet_1.pdf]

Supplementary Material

Supplementary Figures and Tables

Supplementary Table S1. Code-aligned Hodgkin–Huxley model formulation and parameters for all simulated neuronal populations.

**Category / Neuronal Type**	**Equations & Parameters**
1. Synaptic Gating Variables	
AMPAR (PY, TC, RET)	$\frac{dR}{dt}=5\left[1+tanh\left(\frac{V}{4}\right)\right)(1-R)-\frac{R}{2}$
GABAR (LTS, cFS, RE)	$\frac{dR}{dt}=2\left[1+tanh\left(\frac{V}{4}\right)\right)(1-R)-\frac{R}{5A}$
GABAR (sFS, dMSN, iMSN, GPe)	$\frac{dR}{dt}=2\left\{\frac{1}{1+exp[-(V+37)/2)}\right)(1-R)-\frac{0.04R}{A}$
STN AMPAR / GPi GABAR (short-term plasticity)	At an upward crossing of $-10mV$: $u=k\left[5exp(-1)\right)^{-1}r_{dt}$; otherwise $u=0$ $\frac{dR}{dt}=Z$ GPi: $\frac{dZ}{dt}=u-0.4Z-\frac{0.04R}{A}, k=0.30$ STN: $\frac{dZ}{dt}=u-0.4Z-0.04R, k=0.43$
2. General HH Formalism	

Category / Neuronal Type	Equations & Parameters
Gating dynamics (x = m, h, n, etc.)	$\frac{dx}{dt}=\alpha_x(V)(1-x)-\beta_x(V)x$<br>or $\frac{dx}{dt}=\frac{x_\infty(V)-x}{\tau_x(V)}, x=m,h,n,\ldots$
3. Cortical neurons (PY / LTS / cFS)	
Membrane potential	PY: $\frac{dV}{dt}=I_{app}-I_{Na}-I_K-I_{Leak}-I_M+I_{Poisson}-I_{syn}$<br>LTS: $\frac{dV}{dt}=I_{app}-I_{Na}-I_K-I_{Leak}-I_M-I_{syn}$<br>cFS: $\frac{dV}{dt}=I_{app}-I_{Na}-I_K-I_{Leak}-I_{syn}(\text{no}I_M)$<br>$I_{app}\sim N(\mu,\sigma^2=0.1)$<br>$\mu=1.8(\text{PY}), 1.0(\text{LTS}), 0.55(\text{cFS})$
Na kinetics (m, h)	$\alpha_m=\frac{0.32(V+54)}{1-exp[-(V+54)/4)}; \beta_m=\frac{0.28(V+27)}{exp[(V+27)/5)-1}$<br>$\alpha_h=0.128exp[-(V+50)/18); \beta_h=\frac{4}{1+exp[-(V+27)/5)}$
K kinetics (n)	$\alpha_n=\frac{0.032(V+52)}{1-exp[-(V+52)/5)}; \beta_n=0.5exp[-(V+57)/40)$
M-type kinetics (mK; PY/LTS only)	$\alpha_{mK}=\frac{0.0003209(V+30)}{1-exp[-(V+30)/9)}$

Category / Neuronal Type	Equations & Parameters
	$\beta_{mK}=\frac{0.0003209(V+30)}{exp[(V+30)/9)-1}$
Currents	$I_{Na}=50m^3h(V-100); I_K=80n^4(V+100)$<br>$I_{Leak}=0.1(V+61); I_M=2m_K(V+100)$(PY/LTS only)
4. Thalamic Relay (TC)	
Membrane potential	$\frac{dV}{dt}=-I_{Na}-I_K-I_{Leak}-I_{CaT}-I_H-I_{syn}; I_{app}=0$
Na kinetics (m, h)	$\alpha_m=\frac{0.32(V+22)}{1-exp[-(V+22)/4)}; \beta_m=\frac{0.28(V-5)}{exp[(V-5)/5)-1}$<br>$\alpha_h=0.128exp[-(V-2)/18); \beta_h=\frac{4}{1+exp[-(V-5)/5)}$
K kinetics (n)	$\alpha_n=\frac{0.032(V+10)}{1-exp[-(V+10)/5)}; \beta_n=0.5exp[-(V+15)/40)$
T-type Ca (m∞, h)	$m_\infty=\frac{1}{1+exp[-(V+59)/6.2)}; h_\infty=\frac{1}{1+exp[(V+83)/4)}$<br>$\tau_h=\frac{30.8+\frac{211.4+exp[(V+115.2)/5)}{1+exp[(V+86)/3.2)}}{3.73}$
H-current (hF, hS)	$h_{F,\infty}=h_{S,\infty}=\frac{1}{1+exp[(V+68.9)/6.5)}$<br>$\tau_{hF}=exp[(V+183.6)/15.24); \tau_{hS}=\frac{exp[(V+158.6)/11.2)}{exp[(V+75)/5.5)}$
Currents	$I_{Na}=90m^3h(V-50); I_K=80n^4(V+100)$<br>$I_{Leak}=0.01(V+70)+0.0172(V+100)$<br>$I_{CaT}=2m_\infty^2h(V-120); I_H=0.25h_Fh_S(V+40)$
5. Thalamic Reticular (RE)	

Category / Neuronal Type	Equations & Parameters
Membrane potential	$\frac{dV}{dt}=-I_{Na}-I_K-I_{Leak}-I_{CaT}-I_{syn}; I_{app}=0$
Na kinetics (m, h)	$\alpha_m=\frac{0.32(V+42)}{1-exp[-(V+42)/4)}; \beta_m=\frac{0.28(V+15)}{exp[(V+15)/5)-1}$ $\alpha_h=0.128exp[-(V+38)/18); \beta_h=\frac{4}{1+exp[-(V+15)/5)}$
K kinetics (n)	$\alpha_n=\frac{0.032(V+40)}{1-exp[-(V+40)/5)}; \beta_n=0.5exp[-(V+45)/40)$
T-type Ca (m, h)	$m_\infty=\frac{1}{1+exp[-(V+54)/7.4)}; \tau_m=0.44+\frac{0.15}{exp[(V+29)/10)+exp[-(V+104)/15)}$ $h_\infty=\frac{1}{1+exp[(V+82)/5)}; \tau_h=22.7+\frac{0.27}{exp[(V+50)/4)+exp[-(V+409)/50)}$
Currents	$I_{Na}=200m^3h(V-50); I_K=20n^4(V+100)$ $I_{Leak}=0.05(V+90); I_{CaT}=3m^2h(V-120)$
6. Striatal Fast-Spiking (sFS)	
Membrane potential	$\frac{dV}{dt}=-I_{Na}-I_K-I_{Leak}-I_D-I_{gap}-I_{syn}; I_{app}=0$
Na kinetics (m∞, h)	$m_\infty=\frac{1}{1+exp[-(V+24)/11.5)}; h_\infty=\frac{1}{1+exp[(V+58.3)/6.7)}$ $\tau_h=0.5+\frac{14}{1+exp[(V+60)/12)}$
K kinetics (n)	$n_\infty=\frac{1}{1+exp[-(V+12.4)/6.8)}$ $\tau_n=\left\{0.087+\frac{11.4}{1+exp[(V+14.6)/8.6)}\right)\left\{0.087+\frac{11.4}{1+exp[-(V-1.3)/18.7)}\right)$
D-current (m, h)	$m_\infty=\frac{1}{1+exp[-(V+50)/20)}, \tau_m=2$ $h_\infty=\frac{1}{1+exp[(V+70)/6)}, \tau_h=150$

Category / Neuronal Type	Equations & Parameters
Currents	I_{Na}=112.5$m_\infty^3 h(V-50)$; I_K=225$n^2(V+90)$<br>I_{Leak}=0.1$(V+70)$; I_D=6$m^3 h(V+90)$<br>$I_{gap}=\sum 0.1\left(V-V_{pre}\right)$
7. Medium Spiny Neurons (dMSN / iMSN)	
Membrane potential	$\frac{dV}{dt}=-I_{Na}-I_K-I_{Leak}-I_M-I_{CaL}-I_{Kir}-I_{syn}; I_{app}=0$
Na kinetics (m, h)	$\alpha_m=\frac{0.32(V+54)}{1-exp[-(V+54)/4)}; \beta_m=\frac{0.28(V+27)}{exp[(V+27)/5)-1}$<br>$\alpha_h=0.128exp[-(V+50)/18); \beta_h=\frac{4}{1+exp[-(V+27)/5)}$
K and M kinetics (n, M)	$\alpha_n=\frac{0.032(V+52)}{1-exp[-(V+52)/5)}; \beta_n=0.5exp[-(V+57)/40)$<br>$\alpha_M=\frac{0.0003209(V+30)}{1-exp[-(V+30)/9)}; \beta_M=\frac{-0.0003209(V+30)}{1-exp[(V+30)/9)}$
L-type Ca (m, h)	$m_\infty=\frac{1}{1+exp[-(V+33)/6.7)}$<br>$\tau_m=\frac{1}{\frac{0.1194(V+8.124)}{exp[(V+8.124)/9.005)-1}+2.97exp(V/31.4)}$<br>$h_\infty=\frac{1}{1+exp[-(V+13.4)/11.9)}; \tau_h=14.77$
ICaL (implemented GHK flux)	$k=74.87V$<br>$q(V)=\begin{cases} 0, & V\leq -9 \text{or} V=0 \\ 10^{-6}, & -k>80 \\ 2\times10^{-3}, & -k<-80 \\ \frac{2\times10^{-3}-10^{-6}exp(-k)}{1-exp(-k)}, & \text{otherwise} \end{cases}$<br>$I_{CaL}=(4.25\times10^{-7}m^2h)(2\times96489\times V\times k)q(V)$

Category / Neuronal Type	Equations & Parameters
Other currents	$I_{Na}=100m^3h(V-50); I_K=80n^4(V+100); I_{Leak}=0.1(V+67)$<br>$I_M=g_M M(V+100), g_M=2.0\text{(Healthy)or}1.2\text{(PD)}$<br>$I_{Kir}=\frac{g_{Kir}(V+82)}{1+exp[(V+102)/13)}, g_{Kir}=0.175\text{(dMSN)or}0.14\text{(iMSN)}$
8. Globus Pallidus (GPe / GPi)	
Membrane potential	$\frac{dV}{dt}=I_{app}-I_{Na}-I_K-I_{Leak}-I_{CaT}-I_{CaL}-I_{AHP}-I_{syn}$<br>GPe: $I_{app}\sim N(21-13PD,\sigma^2=0.1)$<br>GPi: $I_{app}\sim N(22-6PD,\sigma^2=0.1), PD\in\{0,1\}$
General kinetics (m, h, n)	$m_\infty=\frac{1}{1+exp[-(V+37)/10)}; h_\infty=\frac{1}{1+exp[(V+58)/12)}$<br>$n_\infty=\frac{1}{1+exp[-(V+50)/14)}; \tau_{hn}=0.05+\frac{0.27}{1+exp[(V+40)/12)}$<br>$\frac{dh}{dt}=\frac{0.05(h_\infty-h)}{\tau_{hn}}; \frac{dn}{dt}=\frac{0.1(n_\infty-n)}{\tau_{hn}}$
Ca kinetics (a, r, c)	$a_\infty=\frac{1}{1+exp[-(V+57)/2)}; r_\infty=\frac{1}{1+exp[(V+70)/2)}$<br>$c_\infty=\frac{1}{1+exp[-(V+35)/2)}; \frac{dr}{dt}=\frac{r_\infty-r}{30}$
Ca concentration	$\frac{dCa}{dt}=10^{-4}(-I_{CaT}-I_{CaL}-15Ca)$
Currents	$I_{Na}=120m_\infty^3h(V-55); I_K=30n^4(V+80); I_{Leak}=0.1(V+65)$<br>$I_{CaT}=0.5a_\infty^3r(V-120); I_{CaL}=0.15c_\infty^2(V-120)$<br>$I_{AHP}=\frac{10(V+80)Ca}{Ca+10}$
9. Subthalamic Nucleus (STN)	
Membrane potential	$\frac{dV}{dt}=I_{app}-I_{Na}-I_K-I_{Leak}-I_{CaT}-I_{CaL}-I_{AHP}-I_{syn}+I_{DBS}$<br>$I_{app}\sim N(33-10PD,\sigma^2=0.1), 33\text{(Healthy)}, 23\text{(PD)}$

Category / Neuronal Type	Equations & Parameters
Na/K kinetics (m, h, n)	$m_\infty = \frac{1}{1+exp[-(V+30)/15)}$; $h_\infty = \frac{1}{1+exp[(V+39)/3.1)}$<br>$\tau_h = 1 + \frac{500}{1+exp[(V+57)/3)}$; $n_\infty = \frac{1}{1+exp[-(V+32)/8)}$<br>$\tau_n = 1 + \frac{100}{1+exp[(V+80)/26)}$; $\frac{dh}{dt} = \frac{0.75(h_\infty - h)}{\tau_h}$; $\frac{dn}{dt} = \frac{0.75(n_\infty - n)}{\tau_n}$
T-type Ca (a, b, r)	$a_\infty = \frac{1}{1+exp[-(V+63)/7.8)}$; $b_\infty = \frac{1}{1+exp[-(r-0.4)/0.1)} - \frac{1}{1+exp(4)}$<br>$r_\infty = \frac{1}{1+exp[(V+67)/2)}$; $\tau_r = 7.1 + \frac{17.5}{1+exp[(V+68)/2.2)}$<br>$\frac{dr}{dt} = \frac{0.2(r_\infty - r)}{\tau_r}$
L-type Ca (c)	$c_\infty = \frac{1}{1+exp[-(V+20)/8)}$; $\tau_c = 1 + \frac{10}{1+exp[(V+80)/26)}$<br>$\frac{dc}{dt} = \frac{0.08(c_\infty - c)}{\tau_c}$
Ca concentration	$\frac{dCa}{dt} = 3.75 \times 10^{-5}(-I_{CaT} - I_{CaL} - 22.5Ca)$
Currents	$I_{Na} = 37m_\infty^3 h(V-55)$; $I_K = 45n^4(V+80)$; $I_{Leak} = 2.25(V+60)$<br>$I_{CaT} = 0.5a_\infty^3 b_\infty^2(V-140)$; $I_{CaL} = 2c^2(V-140)$<br>$I_{AHP} = \frac{20(V+80)Ca}{Ca+15}$

Note. Equations were audited against CBGT_loop_model_unparalleled.py, the authoritative 1,573-line model source supplied with this revision. The sensitivity analysis snapshots contain the same 153 neuronal class methods and differ only in fixed-condition, seed, output, and analysis wrappers. V is in mV and time is in ms. A denotes the anesthesia multiplier (A=1 outside propofol and A=4 under propofol). Where the source explicitly provides limiting values at singular voltages for selected Na/K rate expressions, those numerical branches are retained; the table reports the corresponding continuous expressions. The supplied RET generator is called with its default rate of 10 Hz; the internal PD_visual40 identifier is retained only as a legacy file key.

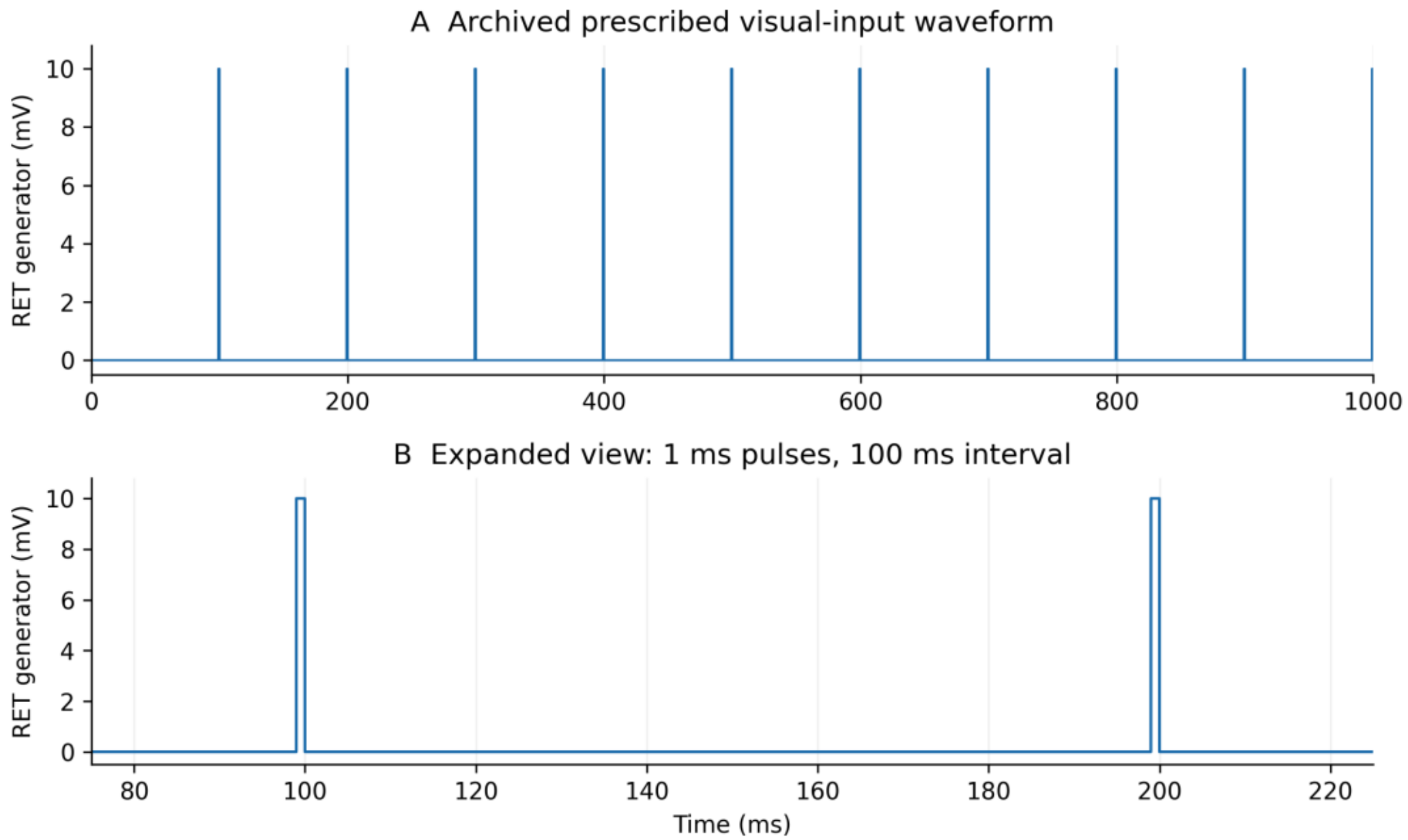

Supplementary Figure S1. Code-audited waveform of the prescribed visual-input generator.

(A) RET generator output over 1,000 ms. The executable creates 1 ms, 10 mV pulses at 100 ms intervals (10 Hz). (B) Expanded view of two consecutive pulses. This waveform was reconstructed directly from Volt_Gen using the archived source constants; no model simulation was rerun.

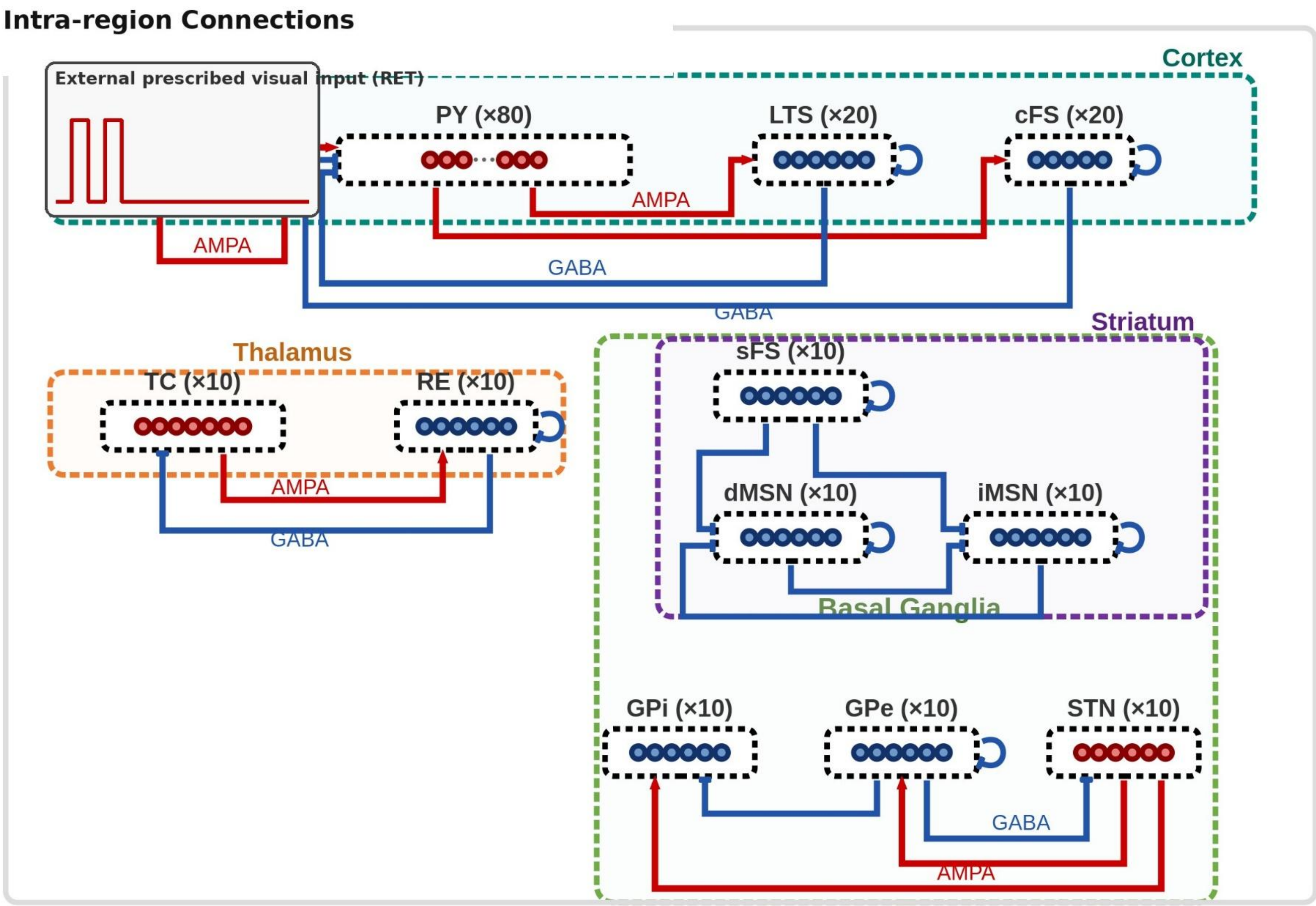

Supplementary Figure S2. Intra-region synaptic connectivity of the CBT network model, shown separately for cortex, thalamus, striatum, and the remaining basal ganglia nuclei. Excitatory (AMPA) projections are drawn in red and inhibitory (GABA) projections in blue; projections that are defined anatomically but not implemented in the model are drawn as dashed lines. Population sizes are given in parentheses. The complete connectivity, including self, lateral, and cross-type projections, is listed in Table 1 of the main text.

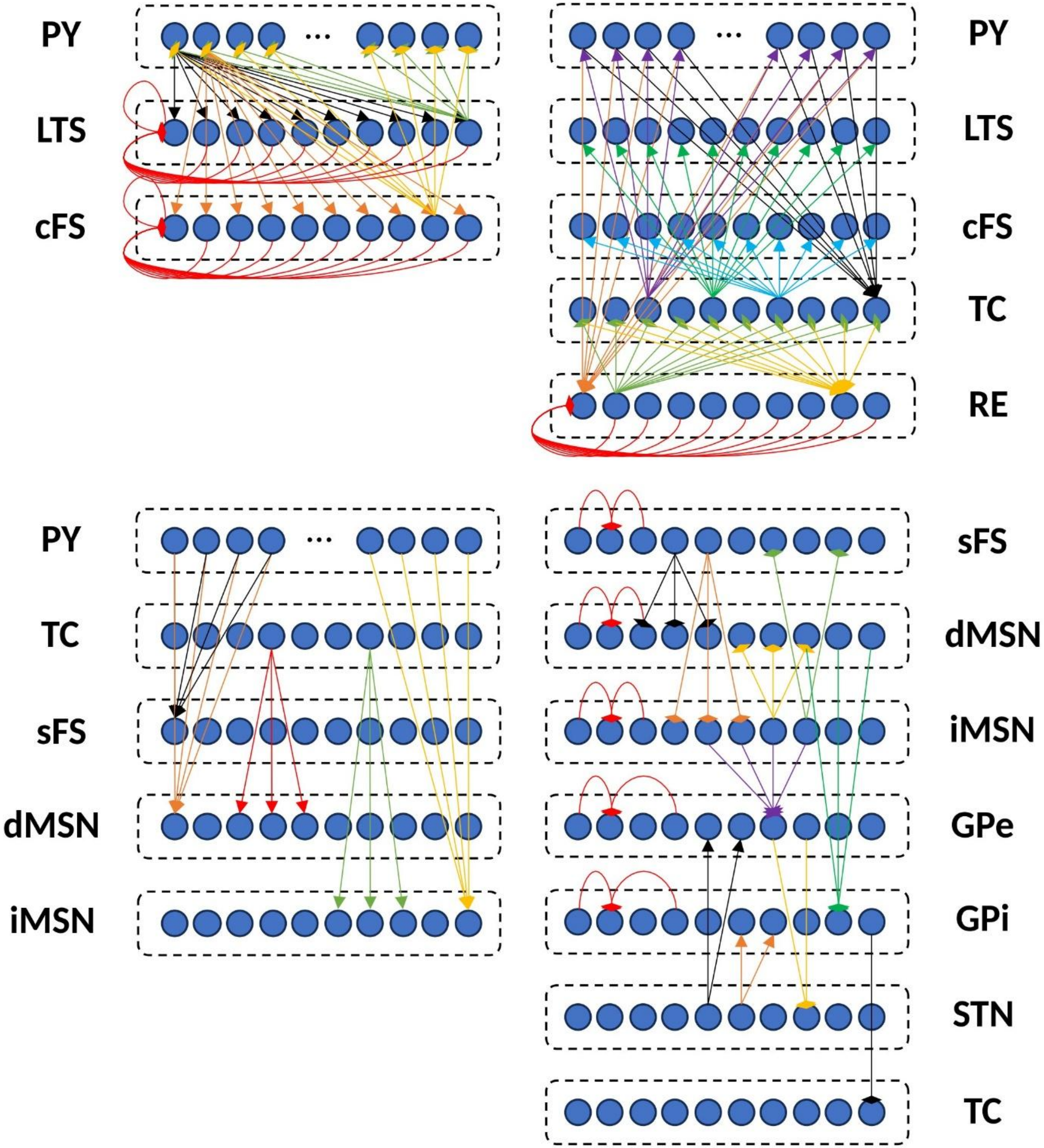
PY
LTS
cFS
PY
LTS
cFS
TC
RE
PY
TC
sFS
dMSN
iMSN
sFS
dMSN
iMSN
GPe
GPi
STN
TC

Supplementary Figure S3. Neuronal composition of each simulated population. The cortical module comprises two structurally identical cortical areas, each containing 40 PY, 10 LTS, and 10 cFS neurons; all thalamic and basal ganglia populations contain 10 neurons each, and RET is a single prescribed input unit.

Supplementary Figures S1–S6 are also provided as separate image files with this submission.

Supplementary Methods S1. Revision analyses

Cortical outputs were calculated from the existing N = 10 run-level files without changing the CBT architecture, state parameters, stimulation waveform, or integration settings. PY firing was obtained from rising-phase crossings through 0 mV. Absolute PY beta, broadband gamma, and narrowband 38–42 Hz power were integrated from the raw postsynaptic-current PSD over 13–30 Hz, 30–80 Hz, and 38–42 Hz, respectively. Planned contrasts used Welch's two-sample t-test with Holm correction within each metric. Time-aligned TC and PY membrane-potential or spike-train series were not jointly retained in the analysis archive; coherence and phase-locking measures could not be reconstructed retrospectively from the retained outputs. DBS-amplitude dependence was evaluated in a dedicated series using 130 Hz biphasic pulses with a 0.06 ms phase width at 0, 1, 2, 3, 4, and 5 mA/cm², with N = 10 independently generated runs per amplitude. All six amplitude groups used the same CBT architecture, 3,000 ms duration, 500 ms transient exclusion, stimulation settings, and raw-PSD pipeline. The groups were not seed matched; the dose summary is therefore an unpaired, between-run descriptive comparison, not a paired within-run dose-escalation experiment. No equivalence test was performed across amplitudes or between the dedicated and primary 5 mA/cm² datasets. One full-resolution TC diagnostic used a dedicated PD+DBS simulation containing 10 TC cells over the 2,500 ms analyzed interval (250,000 native samples per cell) and inspected voltage finiteness, the population-wide voltage range, threshold crossings, and modeled afferent currents. A second, matched-seed local sensitivity analysis varied the model-internal GPi→TC inhibitory coupling coefficient to 0.8, 1.0, and 1.2 × its nominal value of 0.17 (effective coefficients 0.136, 0.170, and 0.204) at 130 Hz, 5 mA/cm², and 0.06 ms per phase. Each scale used the same 10 seeds (2026081800–2026081809). TC voltage was saved for all 10 cells over 500–3,000 ms and decimated from the native 100-kHz integration output to 1 kHz, yielding 250,000 saved samples per scale and 750,000 total. The run was the statistical unit. Raw STN beta was recomputed per run by trapezoidal integration of the un-normalized STN PSD over 13–30 Hz. This one-factor-at-a-time analysis evaluates local robustness at the selected PD+DBS operating point and is not a global, multivariable, or interaction sensitivity analysis. The matched-seed three-parameter local sensitivity analysis varied a GABA_A decay-time-constant factor, the bidirectional STN–GPe core-loop coupling coefficient, and STN injected current one factor at a time to 0.8 and 1.2 × nominal. Each parameter-condition cell used N = 5 shared seeds (2026082400–2026082404), the same 3,000 ms duration and 500 ms transient exclusion, and the same raw-PSD analysis pipeline. M1 was DBS raw STN-beta suppression relative to the single shared nominal PD raw-beta denominator; M2 was spontaneous TC firing under DBS; M3 was the raw STN-beta change under periodic visual input relative to that same nominal PD denominator; and M4 was the

ratio of TC firing under periodic visual input to nominal PD. A separate untreated PD baseline was not rerun for every ±20% parameter level; this was a local one-factor-at-a-time analysis, not a treatment-by-parameter factorial design. Two removable singularities were evaluated with analytic limits, and only the two originally non-finite runs were repeated with their original seeds.

Supplementary Table S2. Cortical PY outputs and planned contrasts (N = 10 per condition).

Metric	Contrast	Group A mean ± SD	Group B mean ± SD	Δ mean	Holm p	Cohen's d
PY firing (Hz)	PD vs Healthy	4.862 ± 0.839	5.830 ± 0.563	−0.968	0.0161	−1.36
PY firing (Hz)	DBS vs PD	5.283 ± 0.867	4.862 ± 0.839	+0.421	0.285	+0.49
PY firing (Hz)	Periodic visual vs PD	10.334 ± 0.852	4.862 ± 0.839	+5.472	1.18×10^{-10}	+6.47
PY beta power	PD vs Healthy	1.360 ± 0.541	1.887 ± 0.989	−0.527	0.483	−0.66
PY beta power	DBS vs PD	1.758 ± 1.013	1.360 ± 0.541	+0.398	0.583	+0.49
PY beta power	Periodic visual vs PD	3.112 ± 0.560	1.360 ± 0.541	+1.752	6.26×10^{-6}	+3.18
PY gamma power (30–80 Hz)	PD vs Healthy	1.623 ± 0.637	1.934 ± 0.368	−0.311	0.403	−0.60
PY gamma power (30–80 Hz)	DBS vs PD	1.706 ± 0.782	1.623 ± 0.637	+0.083	0.798	+0.12

Metric	Contrast	Group A mean ± SD	Group B mean ± SD	Δ mean	Holm p	Cohen's d
PY gamma power (30–80 Hz)	Periodic visual vs PD	7.862 ± 2.001	1.623 ± 0.637	+6.239	6.29×10^{-6}	+4.20
PY 38–42 Hz power	PD vs Healthy	0.125 ± 0.053	0.139 ± 0.088	−0.013	1.000	−0.18
PY 38–42 Hz power	DBS vs PD	0.113 ± 0.039	0.125 ± 0.053	−0.013	1.000	−0.27
PY 38–42 Hz power	Periodic visual vs PD	0.514 ± 0.160	0.125 ± 0.053	+0.389	7.90×10^{-5}	+3.26

Note. Raw powers are in arbitrary units. Holm correction was applied within population and metric across planned contrasts.

Supplementary Table S3. Targeted 130 Hz DBS-amplitude sensitivity (N = 10 per amplitude).

Amplitude (mA/cm²)	STN beta mean	STN beta SD	Change vs 0 (%)	TC firing mean	TC firing SD	PY firing mean	PY firing SD
0	0.02680	0.01577	+0.0	8.268	0.450	5.027	0.651
1	0.05287	0.00753	-97.3	0.000	0.000	5.755	0.977
2	0.00201	0.00037	+92.5	0.000	0.000	5.847	0.686
3	0.00212	0.00064	+92.1	0.000	0.000	6.112	1.243

Amplitude (mA/cm²)	STN beta mean	STN beta SD	Change vs 0 (%)	TC firing mean	TC firing SD	PY firing mean	PY firing SD
4	0.00186	0.00044	+93.1	0.000	0.000	5.668	0.805
5	0.00195	0.00041	+92.7	0.000	0.000	5.652	0.517

Note. Positive change values indicate suppression relative to the 0 mA/cm² group; the −97.3% value at 1 mA/cm² denotes a 97.3% increase in the STN beta measure. STN firing under DBS was not used as a spontaneous-rate endpoint because threshold crossings were stimulation locked.

Supplementary Table S4. Scope of sensitivity evidence and cross-model contextualization.

**Parameter family**	**Direct evidence in the present CBT model**	**Related model or literature context**	**Permitted interpretation**
DBS amplitude	Dedicated, unpaired 0–5 mA/cm² comparison at 130 Hz; N = 10 independently generated runs per level. All six levels used the same architecture, duration, transient exclusion, pulse width, and analysis pipeline. The 1 mA/cm² response was non-monotonic, and mean STN beta values at 2–5 mA/cm² occupied a narrow range.	A prior BGT/DBS implementation from our group examined 0–5000 μA/cm² and 100–185 Hz as continuous control domains (Cho et al., 2024).	Amplitude dependence within the tested calibrated regime; the low value at 5 mA/cm² is not isolated. Equivalence across amplitudes and global robustness are not established.
DBS frequency and temporal pattern	Frequency fixed at 130 Hz; pulse width fixed at 0.06 ms per phase.	Frequency- and protocol-dependent changes are reported in conductance-based BGT models (Rubin and Terman, 2004; Guo et al., 2008; So et al., 2012).	The present study does not establish frequency or waveform robustness.

Parameter family	Direct evidence in the present CBT model	Related model or literature context	Permitted interpretation
GPi→TC inhibition and relay	Matched-seed local GPi→TC analysis at 0.8, 1.0, and 1.2 × nominal coupling (effective coefficients 0.136, 0.170, and 0.204), N = 10 runs per scale at the primary PD+DBS operating point. TC firing remained 0 Hz in 30/30 runs; all 750,000 saved TC voltage samples were finite and subthreshold.	Related models apply explicit sensorimotor pulses and show that relay depends on the temporal pattern of pallidal inhibition (Rubin and Terman, 2004; Guo et al., 2008; So et al., 2012; Cho et al., 2024).	The zero spontaneous-firing output is locally robust to the tested ±20% GPi→TC variation. Evoked relay fidelity and robustness to other parameters or interactions are not established.

Parameter family	Direct evidence in the present CBT model	Related model or literature context	Permitted interpretation
Other coupling, delays, and intrinsic parameters	Outside the targeted GPi→TC and three-parameter one-factor-at-a-time analyses, remaining coupling strengths, delays, intrinsic conductances, and injected currents were fixed at calibrated values.	Hopf analyses show that coupling and delay variations can reorganize oscillatory regimes (Wang et al., 2022; Wang et al., 2023a,b).	Local robustness is supported only for the explicitly tested parameters and ranges; global and interaction robustness remain future work.
Arousal-state GABA/leak parameters	Wake, NREM, and propofol parameter sets are available; PD+propofol was analyzed across N = 10 runs, but interventions were compared only in awake PD.	GABA_A kinetics shape propofol thalamocortical dynamics (Ching et al., 2010); STN low-frequency power changes under propofol (Jiang et al., 2021), and beta can persist in NREM PD (Mizrahi-Kliger et al., 2020).	State configurability is supported; intervention-by-arousal invariance is not established.
GABA_A decay, STN–GPe coupling, and STN	Matched-seed one-factor-at-a-time ±20% analysis; N = 5 shared seeds per parameter-condition cell. M1–M4	The reviewer-nominated parameter classes were varied directly in the present CBT implementation.	All four outcomes persisted within these tested local ranges. Treatment-by-parameter interactions

Parameter family	Direct evidence in the present CBT model	Related model or literature context	Permitted interpretation
injected current	used raw PSD and a single shared nominal PD denominator (Supplementary Table S7).		and multivariable robustness were not tested.

Note. Evidence from related models is used for parameter provenance and cross-model contextualization; it is not presented as empirical sensitivity analysis of the current CBT implementation.

Supplementary Table S5. Diagnostic interpretation of zero spontaneous TC firing under DBS.

Diagnostic item	Existing evidence and interpretation
Amplitude-series output	TC spontaneous firing was 0 ± 0 Hz at each of 1, 2, 3, 4, and 5 mA/cm² (N = 10 per level; 50 stimulated run-level outputs).
Dedicated diagnostic simulation	One PD+DBS simulation containing all 10 modeled TC cells over the 2,500 ms analyzed interval; 250,000 voltage samples per cell.
Voltage-data integrity	2,500,000 of 2,500,000 samples were finite; NaN count = 0.
Population-wide voltage range	−71.100 to −57.925 mV; no sample crossed the 0-mV firing threshold.
Modeled afferent currents	PY→TC excitation and GPi→TC inhibition were nonzero. RET was inactive because no visual drive was applied; RE output was silent in this resting DBS condition.

Diagnostic item	Existing evidence and interpretation
Supported interpretation	In the dedicated diagnostic run, finite subthreshold voltages are inconsistent with sustained depolarization block and are most consistent with spontaneous quiescence under the calibrated DBS/input balance.
GPi→TC connectivity sensitivity (10 runs × 3 scales)	At 0.8, 1.0, and 1.2 × nominal GPi→TC coupling (N = 10 matched-seed runs per scale), TC spontaneous firing was 0 Hz in 30/30 runs. Across 750,000 saved voltage samples, the overall range was −72.058 to −54.742 mV; no sample exceeded −30 mV and no 0-mV crossing occurred. Run-mean voltage became progressively more hyperpolarized as the coupling coefficient increased. These data are consistent with subthreshold quiescence across the tested local range.
Not established	Evoked sensorimotor relay fidelity, blockade of all information transmission, global robustness, and robustness to other parameters or combined perturbations.

Note. The full-resolution diagnostic comprised one run with 2.5 million native voltage samples. The matched-seed local GPi→TC analysis comprised N = 10 runs per scale with all 10 TC cells saved at 1 kHz over 500–3,000 ms (250,000 samples per scale; 750,000 total). The statistical unit for between-scale summaries was the run, not the cell or voltage sample.

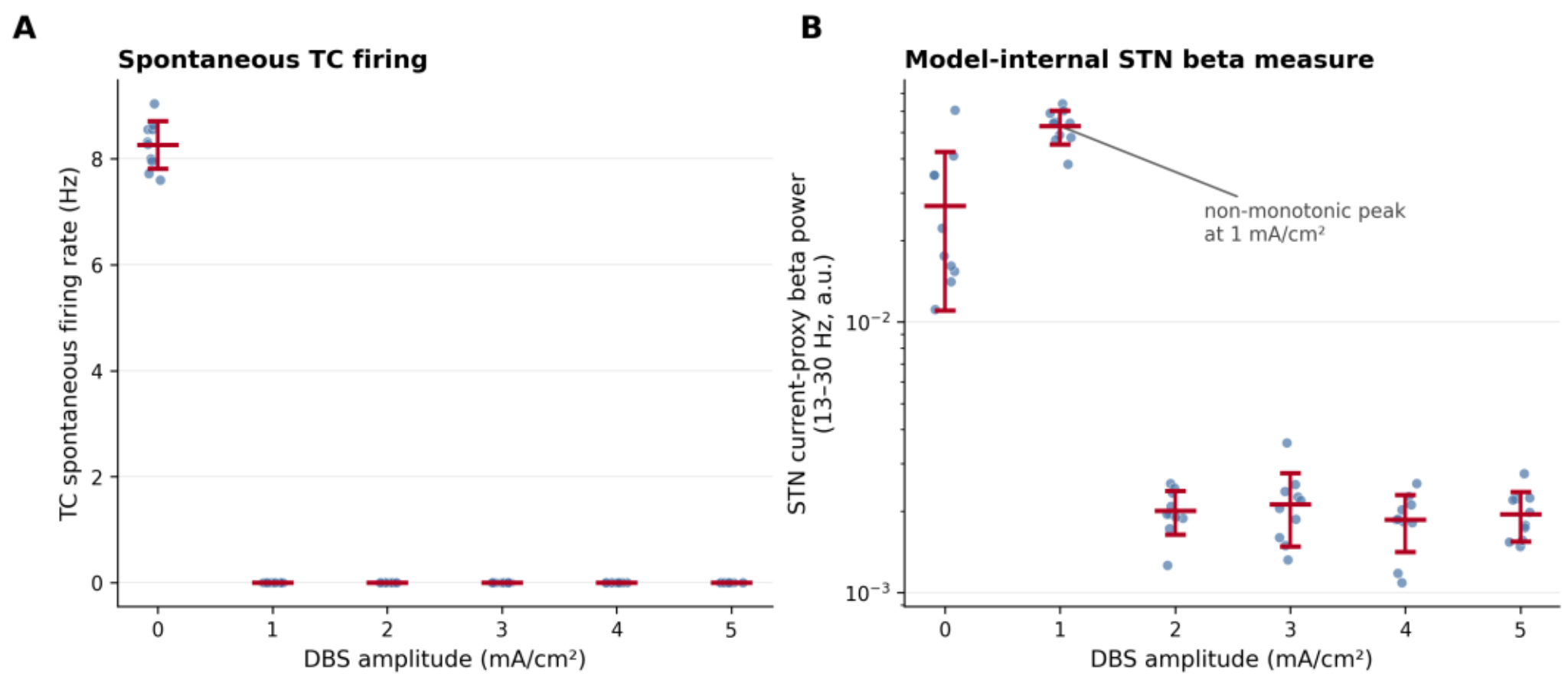

Supplementary Figure S4. Targeted, unpaired 130 Hz DBS-amplitude series in the current CBT model (N = 10 independently generated runs per amplitude). (A) Spontaneous TC firing. (B) Model-internal STN outgoing-current beta measure (13–30 Hz). Blue points denote individual stochastic runs and red bars denote mean ± SD. All six amplitude groups (0–5 mA/cm²) were generated in the same dedicated series. TC spontaneous firing was 0 Hz in all 50 stimulated run-level outputs. The STN beta measure increased non-monotonically at 1 mA/cm² and was lower at 2–5 mA/cm². These results characterize amplitude dependence in the tested calibrated regime and do not measure evoked relay fidelity. Local GPi→TC sensitivity is reported separately in Supplementary Figure S5 and Supplementary Tables S5–S6.

Supplementary Table S6. Matched-seed local GPi→TC sensitivity at the primary PD+DBS operating point.

Scale	Effective coefficient	N	TC firing (Hz)	Run-mean TC voltage (mV)	Pooled voltage range (mV)	0-mV crossings	Raw STN beta (a.u.)
0.8×	0.136	10	0.000 ± 0.000	−67.227 ± 0.035	−70.332 to −57.786	0	0.001931 ± 0.000333
1.0×	0.170	10	0.000 ± 0.000	−67.903 ± 0.038	−71.269 to −56.334	0	0.001931 ± 0.000333
1.2×	0.204	10	0.000 ± 0.000	−68.497 ± 0.039	−72.058 to −54.742	0	0.001931 ± 0.000333

Note. Values are mean ± SD across runs unless identified as pooled ranges. Voltage ranges combine all 250,000 saved samples per scale for diagnostics; the inferential unit remains the run. No equivalence test or multivariable sensitivity analysis was performed.

Supplementary Table S7. Matched-seed ±20% one-factor-at-a-time local sensitivity of a GABA_A decay factor, STN–GPe coupling conductance, and STN injected current.

Parameter	Factor	M1: raw DBS β-suppression	M2: TC firing (DBS)	M3: raw visual-condition β-change	M4: visual-condition TC fold	Finite runs
Nominal	1.0	94.2%	0 Hz (5/5)	+76.2%	4.19×	5/5
GABA_A τ	0.8	93.5%	0 Hz (5/5)	+121.5%	4.07×	5/5
GABA_A τ	1.2	92.9%	0 Hz (5/5)	+83.6%	4.20×	5/5
STN–GPe coupling	0.8	95.8%	0 Hz (5/5)	+216.6%	3.70×	5/5
STN–GPe coupling	1.2	88.9%	0 Hz (5/5)	+102.1%	4.29×	5/5
STN I_app	0.8	92.3%	0 Hz (5/5)	+177.0%	4.27×	5/5
STN I_app	1.2	92.4%	0 Hz (5/5)	+230.9%	3.81×	5/5

Note. Each perturbation used five shared stochastic seeds (2026082400–2026082404). M1–M2 were evaluated under PD+DBS and M3–M4 under PD+periodic visual input. M1 and M3 use trapezoidal integration of raw STN PSD over 13–30 Hz and the same single nominal PD denominator; a separate untreated PD baseline was not rerun for each ±20% parameter level. All 15 parameter-condition cells contained five finite runs. All outcomes were preserved (M1 = 88.9–95.8%, M2 = 0 Hz, M3 = +76.2% to +230.9%, M4 = 3.70–4.29×). Two removable singularities were stabilized analytically and only the two affected runs were repeated with their original seeds. This is a targeted local analysis; combined-parameter, delay, and waveform variations are not tested.

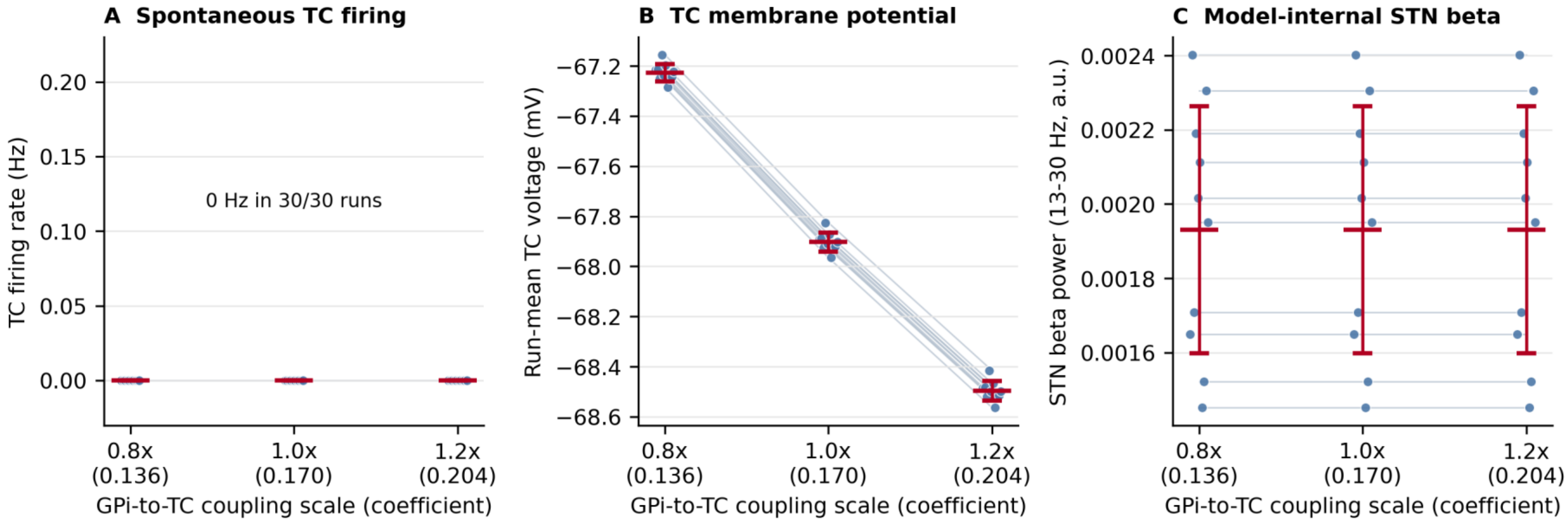

Supplementary Figure S5. Matched-seed one-factor-at-a-time sensitivity of the model-internal GPi→TC inhibitory coupling coefficient at the primary PD+DBS operating point (130 Hz, 5 mA/cm², 0.06 ms per phase; N = 10 runs per scale). (A) Spontaneous TC firing. (B) Run-mean TC membrane potential. (C) Raw STN outgoing-current beta power integrated over 13–30 Hz. Thin lines connect identical stochastic seeds across 0.8, 1.0, and 1.2 × nominal coupling; blue points are run-level values and red bars are mean ± SD. TC firing was 0 Hz in 30/30 runs. All 750,000 saved TC voltage samples were finite and subthreshold, whereas raw STN beta was numerically identical across the three scales because identical seeds and persistent TC silence produced no scale-dependent feedback difference to the upstream STN signal. These results show persistence across the tested local GPi→TC range but do not establish global or interaction sensitivity or evoked relay fidelity.

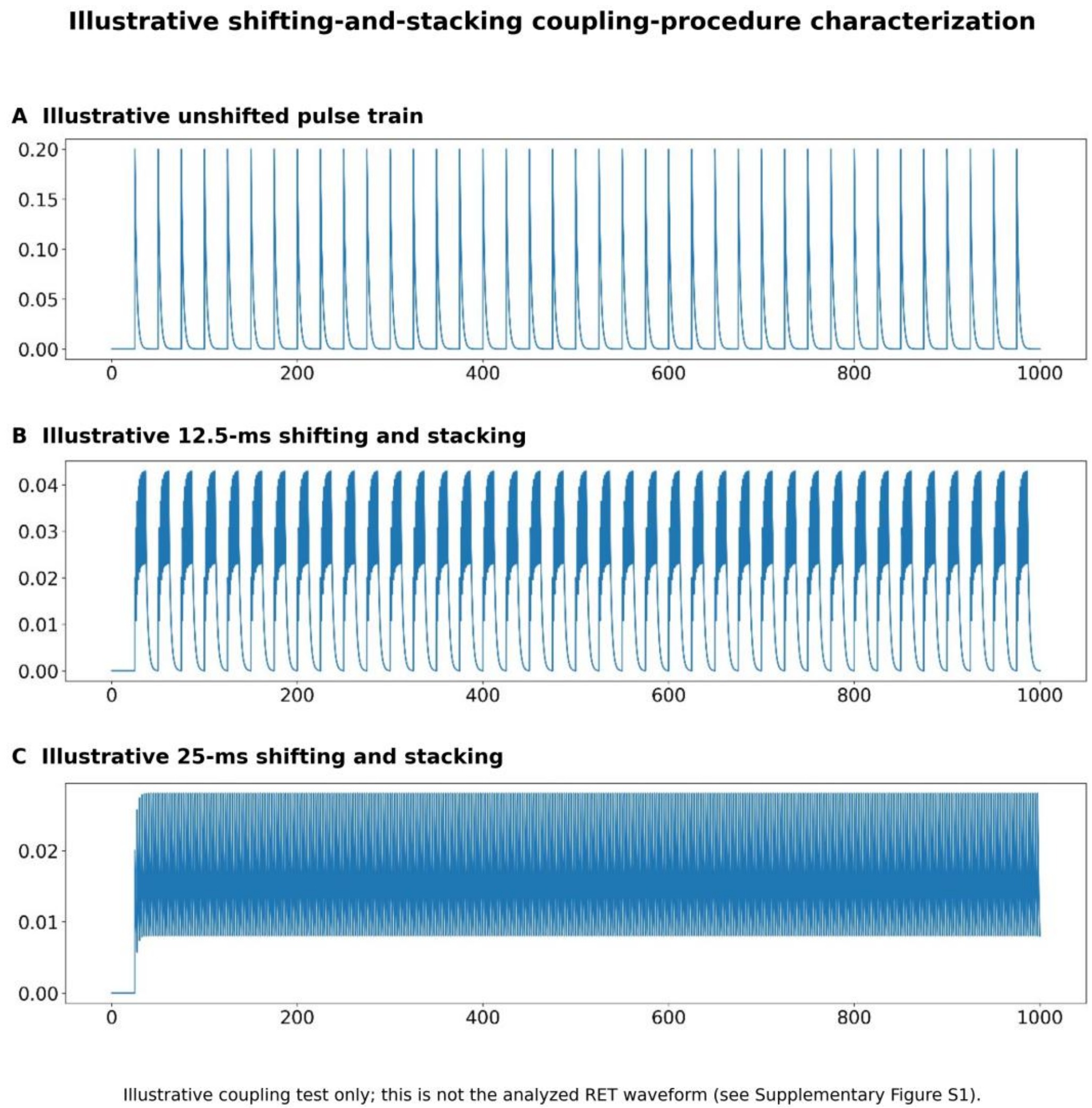

Supplementary Figure S6. Illustrative shifting-and-stacking coupling-procedure characterization. (A) Unshifted pulse train. (B,C) Stacking after 12.5- and 25-ms uniform shifts. These archived panels test numerical signal compatibility only; their pulse spacing is illustrative and is not the analyzed RET waveform, a 40-Hz stimulation condition, or evidence of phase entrainment. The code-audited 10-Hz RET waveform is shown in Supplementary Figure S1. The procedure is an engineering approximation, not a biological axonal-delay model.
